# Bacteriophage Crosstalk: Coordination of Prophage Induction by Trans-Acting Antirepressors

**DOI:** 10.1371/journal.pgen.1002149

**Published:** 2011-06-23

**Authors:** Sébastien Lemire, Nara Figueroa-Bossi, Lionello Bossi

**Affiliations:** Centre de Génétique Moléculaire, CNRS, UPR3404, Université Paris-Sud, Gif-sur-Yvette, France; Universidad de Sevilla, Spain

## Abstract

Many species of bacteria harbor multiple prophages in their genomes. Prophages often carry genes that confer a selective advantage to the bacterium, typically during host colonization. Prophages can convert to infectious viruses through a process known as induction, which is relevant to the spread of bacterial virulence genes. The paradigm of prophage induction, as set by the phage Lambda model, sees the process initiated by the RecA-stimulated self-proteolysis of the phage repressor. Here we show that a large family of lambdoid prophages found in *Salmonella* genomes employs an alternative induction strategy. The repressors of these phages are not cleaved upon induction; rather, they are inactivated by the binding of small antirepressor proteins. Formation of the complex causes the repressor to dissociate from DNA. The antirepressor genes lie outside the immunity region and are under direct control of the LexA repressor, thus plugging prophage induction directly into the SOS response. GfoA and GfhA, the antirepressors of *Salmonella* prophages Gifsy-1 and Gifsy-3, each target both of these phages' repressors, GfoR and GfhR, even though the latter proteins recognize different operator sites and the two phages are heteroimmune. In contrast, the Gifsy-2 phage repressor, GtgR, is insensitive to GfoA and GfhA, but is inactivated by an antirepressor from the unrelated Fels-1 prophage (FsoA). This response is all the more surprising as FsoA is under the control of the Fels-1 repressor, not LexA, and plays no apparent role in Fels-1 induction, which occurs *via* a Lambda CI-like repressor cleavage mechanism. The ability of antirepressors to recognize non-cognate repressors allows coordination of induction of multiple prophages in polylysogenic strains. Identification of non-cleavable *gfoR*/*gtgR* homologues in a large variety of bacterial genomes (including most *Escherichia coli* genomes in the DNA database) suggests that antirepression-mediated induction is far more common than previously recognized.

## Author Summary

Many viruses that infect bacteria (bacteriophages) can direct the integration of their DNA into the bacterial chromosome. This condition, known as lysogeny, is relevant to bacterial evolution, as it is one of the main pathways leading to the incorporation of foreign DNA in nature. Indeed, bacteriophages often carry genes that escape lysogenic repression and benefit the bacterium. This symbiotic association can come to an end if bacteria suffer DNA damage. A mechanism mediated by the host's RecA protein causes the relief of repression, viral DNA excision, and replication. This process, known as prophage induction, kills the host and results in the release of viral particles. In this work, we have analyzed the mechanism responsible for induction in a large family of prophages naturally present in the genomes of *Salmonella* bacteria. We found that, unlike in best-studied model phages, the repressors of these *Salmonella* phages do not undergo RecA-mediated proteolysis; rather, they are inactivated by the binding of small antirepressor proteins. We show that some antirepressors can act on both cognate and non-cognate repressors, allowing separate prophages within a given strain to be induced simultaneously. We discuss evidence suggesting that antirepressor-mediated prophage induction is quite common in the bacterial world.

## Introduction

Temperate bacteriophages are major players in the evolution of bacterial genomes. Phages can act as vectors for gene transfer and, by virtue of their ability to integrate in the bacterial chromosomes, they can permanently modify the properties of the host cell. Such “lysogenic conversion” is particularly prominent in enteric bacteria presumably due to their promiscuous lifestyle. Enteric species like *E. coli* and *Salmonella* typically contain multiple resident prophages whose variability in number and assortment constitutes a major source of diversity between strains [1]–[3]. Some prophages express functions that contribute to pathogenicity. Lysogenization of *E. coli* by bacteriophages carrying Shiga-like toxin genes converts a harmless commensal into a dreadful enteric pathogen [4]. The toxin gene *stx* is repressed in the lysogenic state, but is activated under conditions that elicit prophage induction [5], [6]. In *Salmonella*, the contribution of prophages to pathogenicity results from the synergistic action of multiple factors playing subtle and often redundant roles. The genes encoding such factors are expressed in the lysogenic state under the control of the regulatory circuitry of the host bacterium [7], [8].

Gifsy-1 and Gifsy-2 are lambdoid prophages found in most strains of *Salmonella enterica* serovar Typhimurium and were originally identified genetically during a study of *recB* suppressor mutations in strain LT2 [9]. Both phages contain *recET* gene orthologs that, although repressed in the lysogenic state, can be activated by mutation, resulting in the suppression of recombination defects. A third Gifsy-related prophage found in another model strain, ATCC14028, has been named Gifsy-3 [2]. All three prophages exhibit the typical modular organization of bacteriophage λ with two identifiable divergent transcription units originating from a site roughly one third away from the left end of the prophage map [8], [10]. When induced, all three prophages form virions that closely resemble λ [11]. As the genome sequences from an increasing number of serovar Typhimurium strains have become available, it has become possible to compare the sequences of their resident Gifsy prophages. This analysis revealed that Gifsy-1 displays extensive polymorphism in the region surrounding the lysogenic repressor and other regulatory elements [7], [8]. Conversely, Gifsy-2 is highly conserved throughout the serovar, while Gifsy-3 appears to be a specific acquisition of strain ATCC14028 [2], [12].

Phage circulation among strains results from conditions that relieve lysogenic repression and elicit the developmental program of the virus. The paradigm for this induction process is set by widely studied phages such as λ and P22. In both of these phages, induction results from the autocatalytic cleavage of a repressor triggered by the accumulation of RecA-DNA filaments [13], [14]. λ and P22 repressor proteins, 237 and 216 amino acids (aa), respectively, contain two domains: an N-terminal DNA-binding domain and a C-terminal oligomerization domain with the cleavage activity [15]. RecA-stimulated cleavage occurs at identical alanyl-glycil sequences near the center of both proteins [16] and is catalyzed by a highly conserved Lys/Ser dyad. Identification of the Gifsy-1/-2 phage repressor in strain LT2 revealed it to be significantly smaller (136 aa) than the λ or P22 repressors and to lack the signature motif for autocatalytic cleavage [10]. Examination of the Gifsy prophage sequences from other strains showed them to have similar small sizes, raising the question of the mechanism responsible for repressor inactivation in these prophages. The work described in this paper was aimed at answering this question. We show that the induction of Gifsy prophages does not result from repressor cleavage, but rather from repressor inactivation consequent to the binding of antirepressor proteins. The genes encoding these antirepressors are located outside the immunity region and under direct control of the LexA protein. A similar regulatory mechanism was previously described in coliphages 186 and N15 [17], [18]. Interestingly, some of the antirepressors identified here have the ability to act on non-cognate repressors, providing the basis for a molecular crosstalk that allows coordinating the induction of multiple prophages in polylysogenic bacteria.

## Results

### Variability of Gifsy phage repressors

**Figure 1:**
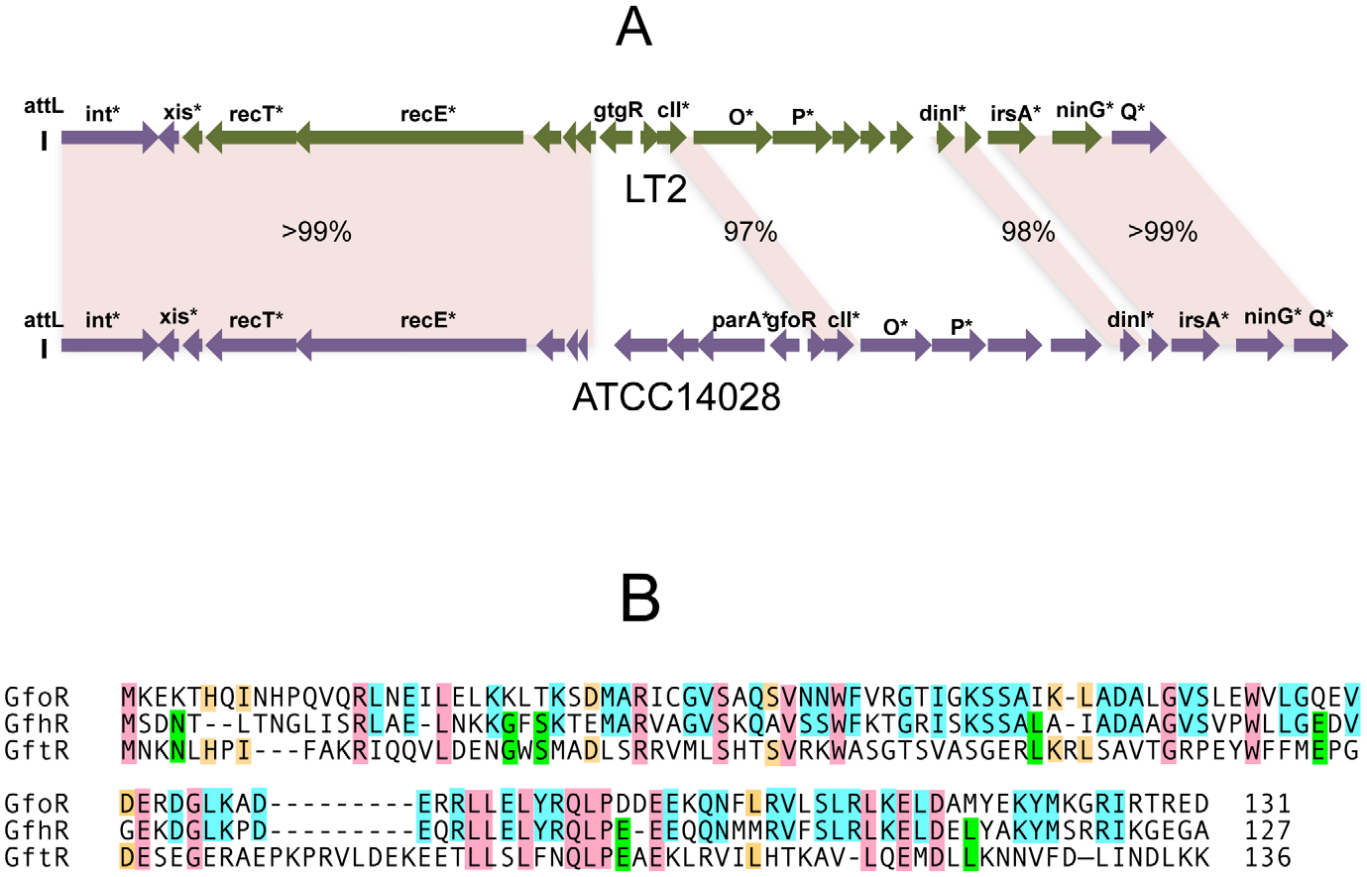
Variability of Gifsy phage repressors. A. Comparison of the Gifsy-1 prophage genomes from *Salmonella enterica* serovar Typhimurium strains LT2 and ATCC14028. Diagrams were made from sequence data obtained in the course of this study, complemented with data from [21] and [12]. Percentages indicate DNA sequence identities. Green coloring shows a portion of LT2's Gifsy-1 prophage more than 99% identical to the corresponding region of Gifsy-2. Genes marked by an asterisk are named on the basis of their sequence similarity to known genes of other phages or bacteria. B. Alignment of the deduced amino acid sequences of the repressors of prophages Gifsy-1 (GfoR) Gifsy-3 (GfhR) and Gifsy-2 (GftR) from strain ATCC14028.

In strain LT2, an approximately 12 Kb portion of the Gifsy-2 prophage genome, including the immunity region together with replication and recombination functions, is duplicated at the corresponding position of the Gifsy-1 genome [10]. Conceivably, a recombination or conversion event homogenized the two sequences during the evolutionary history of this strain. As a result, the Gifsy-1 repressor of LT2, GogR, is a perfect copy of the Gifsy-2 repressor, GtgR, and the two phages are homoimmune [7], [10]. Sequence analysis of the Gifsy phages in strain ATCC14028 showed the immunity region of Gifsy-1 to differ extensively from that of Gifsy-1/-2 prophages of strain LT2 (Figure 1A). In contrast, Gifsy-2 sequences are nearly identical in the two strains, while Gifsy-3 carries a different immunity region. The presumptive repressor genes of the Gifsy-1, and Gifsy-3 prophages of strain ATCC14028 were named *gfoR*, and *gfhR*, respectively. GfoR (Gifsy-1) and GfhR (Gifsy-3) share 66.4% similarity in their amino acid sequences and are 32,6% and 33,8% similar to GftR (Gifsy-2), respectively (Figure 1B). The sequence of the latter is 100% identical to that of LT2's GtgR. Finally, it is worth mentioning that Gifsy-3 repressor, GfhR, is 100% identical to the repressor of a prophage found at the site of Gifsy-1 in strain SL1344, another *Salmonella* model strain [19](GenBank FQ312003). These findings account for the original observation that ATCC10028's Gifsy-3 and SL1344's Gifsy-1 phages are homoimmune [7] and provide further evidence for extensive module shuffling between *Salmonella* phages.

### Gifsy prophage repressors are not cleaved during induction

**Figure 2:**
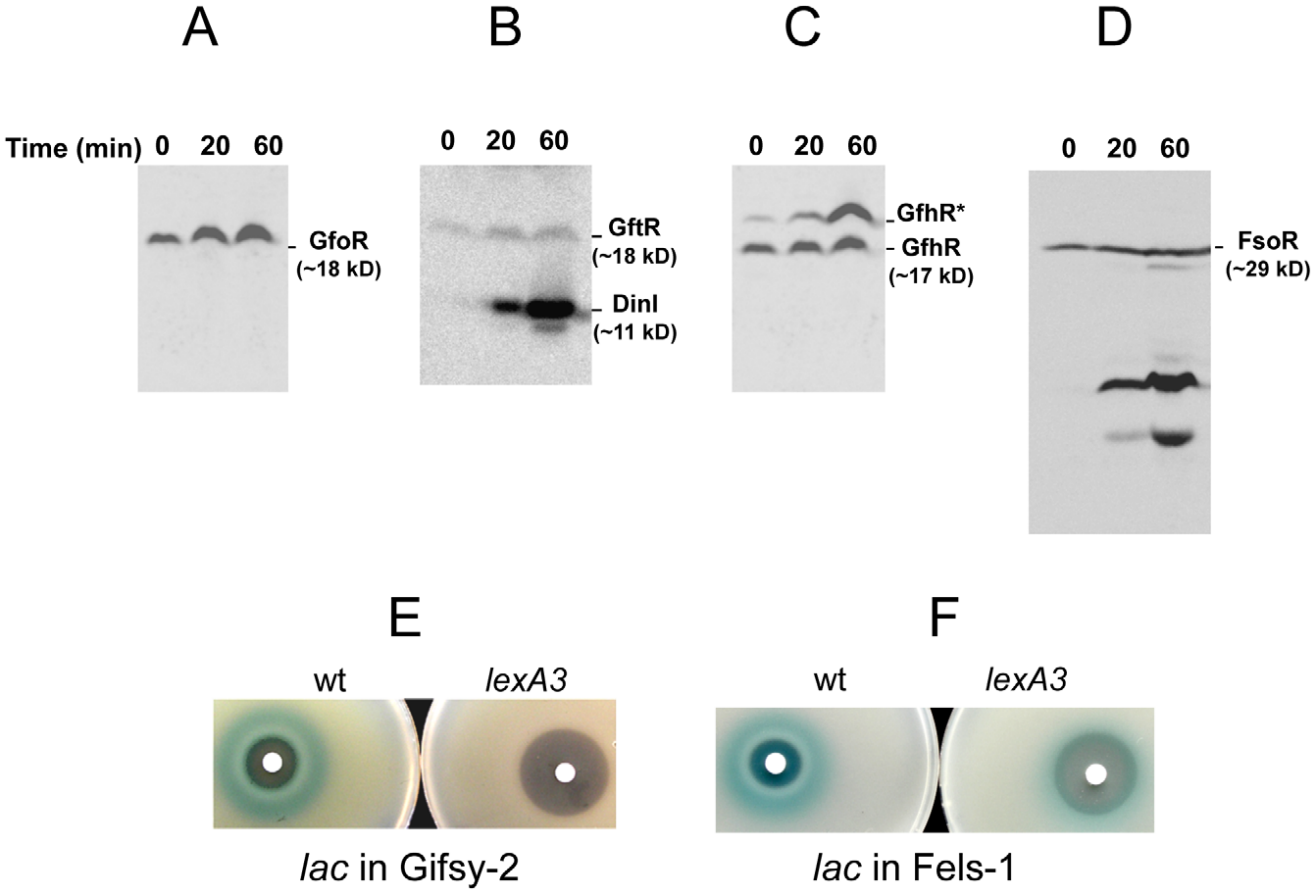
Fate of phage repressors during induction and the role of LexA. Strains harboring C-terminally 3xFLAG-tagged versions of the repressors of prophages Gifsy-1 (A) Gifsy-2 (B), Gifsy-3 (C) and Fels-1 (D) were exposed to Mitomycin C (1 µg mL^−1^) for the indicated times and processed for immunodetection of the 3xFLAG epitope as described [20]. Strains used were MA8407 (A), MA8259 (B), MA8408 (C) and MA8456 (D). E,F. Effect of the *lexA3* mutation on induction of *lacZ* reporter fusions in the Gifsy-2 prophage (E) or in the Fels-1 prophage (F). Cultures were spread on LB X-gal indicator plates; filter paper disks were placed on the surface and soaked with 5 µL of 2 mg mL^−1^ Mitomycin C. The strains used were MA8756 (*lexA* ^+^) and MA8757 (*lexA3*) in E and MA8410 (*lexA* ^+^) and MA8573 (*lexA3*) in F. For complete strain genotypes, see Table 1.

To monitor the fate of Gifsy prophage repressors under inducing conditions, variants of the *gfoR*, *gftR* and *gfhR* genes carrying carboxy-terminal 3xFLAG epitope tags were constructed in the ATCC14028 chromosome [20]. Tagged GfoR and GfhR remained competent to confer immunity against the corresponding phage (data not shown), suggesting that presence of the tag did not adversely affect the function of the proteins. Similar epitope tag fusions were derived from two additional genes: a *dinI* homologue in the Gifsy-2 left operon, to serve as a control for the transcriptional response to the inducing treatment, and a gene presumed to encode the repressor of the Fels-1 prophage of strain LT2 [21]. Fels-1 putative repressor, hereafter referred to as FsoR, is a 231 aa protein similar to λ's CI repressor and thus expected to undergo cleavage during induction. Exponentially growing cells from strains carrying the 3xFLAG-tagged genes were exposed to Mitomycin C (MitC) and processed for Western blot detection using anti-FLAG antibodies. As shown in Figure 2, none of the Gifsy repressors suffers detectable cleavage throughout the treatment, while the accumulation of the DinI protein (Figure 2B), together with the appearance of cleavage products of the FsoR repressor (Figure 2D), confirm that induction is taking place. In Gifsy-3, the MitC treatment leads to the accumulation of a GfhR variant with an N-terminal extension (marked GfhR* in Figure 2C). This protein originates from an upstream, in-frame AUG codon (Figure S1). A construct with the longer open reading frame fused to the P^BAD^ promoter expressed the shorter version of GfhR in the absence of arabinose (Figure S1) suggesting that the *gfhR* promoter lies within the interval between the two AUGs. GfhR* must therefore originate from a different promoter, located upstream from the primary promoter, and apparently activated upon induction. The role of GfhR*, if any, was not further investigated here.

### Gifsy prophage induction requires activation of a LexA-regulated locus outside the immunity region

The results in Figure 2 suggested that induction of the Gifsy prophages in strain ATCC14028 occurs by a mechanism not involving cleavage of repressors. Experiments with *lacZ* fusions supported this conclusion. Fusions of *lacZ* to the *recE* gene, or to an homologue of λ's *cII* gene were no longer activated by MitC when combined with deletions that remove material to the right of the DNA replication genes (see diagram in Figure S2). Thus, the induction mechanism requires one or more genes located outside of, and relatively distant from, the immunity region. A previous report identified potential LexA binding sites in Gifsy prophage genomes [22]. To assess the role of LexA in Gifsy induction, we made use of the *lexA3* allele, which produces a non-cleavable form of the LexA protein [23]. The mutation was introduced into strains with *recE*-*lacZ* fusions in either Gifsy-1 or Gifsy-2 and the resulting strains were tested for their response to MitC on X-gal indicator plates. As shown for Gifsy-2 in Figure 2E, *lexA3* completely abolishes MitC-dependent induction. Thus, LexA cleavage appears to be required for Gifsy prophage induction. In contrast, the *lexA3* mutation does not prevent the MitC-dependent activation of a *lacZ* fusion in the late operon of the Fels-1 prophage (Figure 2F). The latter findings are consistent with the idea that Fels-1 induction results directly from cleavage of a CI-type repressor (Figure 2D).

**Figure 3:**
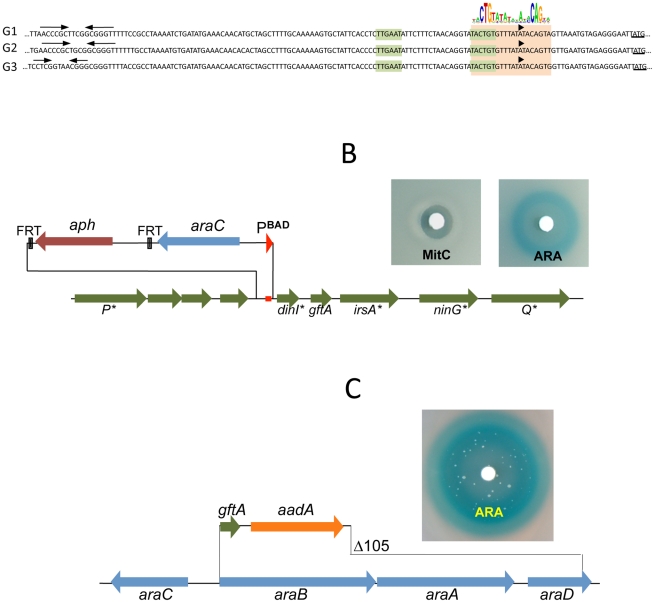
LexA-controlled prophage loci required for induction. A. Alignment of DNA sequences preceding the *dinI* gene homologues of Gifsy-1, Gifsy-2 and Gifsy-3 prophages. The *dinI* translation initiation codon is underlined. The −35 and −10 motifs of putative promoters are highlighted in light green. A light brown box encompasses sequences matching the consensus for LexA binding. B. Effect of replacing the LexA box of the Gifsy-2 prophage with an araC-P^BAD^ promoter module on induction of a Gifsy-2-borne *recE-lacZ* fusion (strain MA8357). C. Expression of Gifsy-2's *gftA* gene alone (strain MA8430) is sufficient to elicit induction of the prophage.

The presumptive LexA binding site lies within the region of sequence identity between Gifsy-1 and Gifsy-2 prophages of strain LT2. The site is located 1.3 Kb downstream from the replication genes and adjacent to the *dinI* gene homologue. The LexA box is also found at the corresponding position for all three Gifsy prophages of strain ATCC14028, in all instances preceded by palindromic sequences resembling Rho-independent transcription terminators (Figure 3A). To assess the requirement of the LexA binding motif for regulation, the segment between the putative terminator and the AUG translation initiation codon of the *dinI* homologue in the Gifsy-2 prophage was deleted and replaced with an *araC*-P^BAD^ promoter module. The construct was combined with a *recE-lacZ* translational fusion in an LT2-derived strain cured for Gifsy-1. Disk tests on Lac indicator plates showed that the promoter replacement completely abolishes MitC-dependent activation and renders the *recE-lacZ* fusion inducible by arabinose (Figure 3B). Thus, these results confirmed the existence of an SOS locus within the Gifsy-2 genome and suggested that this locus includes one or more genes needed for induction.

### Small antirepressor proteins responsible for Gifsy prophage induction

The analysis of nested deletions originating at the right end of the prophage allowed delimiting the minimal sequence required for *recE*-*lacZ* activation to the interval between the *dinI* and *irsA* homologues (Figure S2C). The region could encode a small protein starting from a non-canonical GUG codon. To assess the role of this locus in prophage induction, the ORF sequence was moved under the control of the chromosomal P^BAD^ promoter, starting with an AUG codon corresponding to the initiation codon of the *araB* gene. Exposure of the resulting strain to a paper disk soaked with arabinose produced a halo of bacterial killing around the disk concomitant to the release of ß-galactosidase activity from the lysed cells (Figure 3C). Together, these effects suggested that the sequence being analyzed contained all the information needed to elicit prophage induction. Interestingly, a fortuitous single bp insertion near the 5′ end of the sequence completely abrogated arabinose-dependent killing and *lac* expression. Since the mutation alters the reading frame of the putative gene, these findings strongly suggested that the inducing molecule was a protein as opposed to an RNA. We postulated that this protein acts as an antirepressor and named it GftA (Gifsy-two antirepressor). As seen in Figure 3C, colonies appeared in the area of bacterial lysis upon prolonged incubation. Characterization of a number of these arabinose-resistant isolates showed some of them to result from prophage deletions while others carried mutations linked to the *ara* locus. One class of mutants had changes in the *araC* gene. Presumably these mutations affect the ability of the AraC protein to bind arabinose thus preventing P^BAD^ promoter activation. A second class of mutation fell within the *gftA* coding sequence and tentatively identified residues important for antirepressor function (see below).

**Figure 4:**
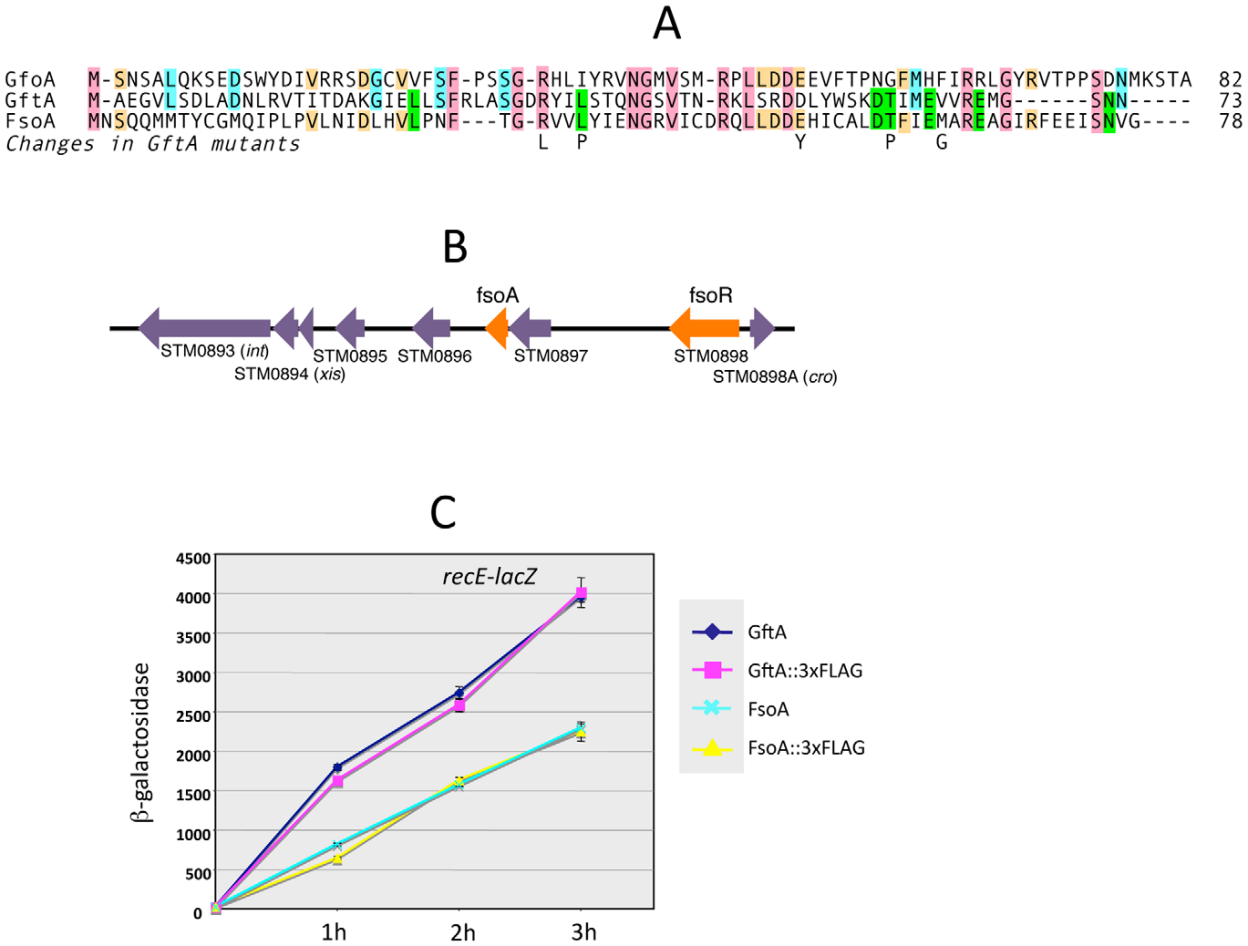
Trans-acting antirepressors. A. Alignment of antirepressor sequences and amino acid changes in GftA mutants. B. Gene organization near the left end of the Fels-1 prophage. The diagram in B was drawn using information from *Salmonella enterica* serovar Typhimurium strain LT2 genome sequence [21]. Repressor and antirepressor genes (*fsoR* and *fsoA*, respectively) were identified in the course of this study. C. Induction of Gifsy-2-borne *recE-lacZ* fusion in strains carrying *gftA* or *fsoA* genes, or their 3xFLAG-tagged variants, fused to the chromosomal P^BAD^ promoter. Arabinose (10 mM) was added at time zero. Cells collected at the indicated times were assayed for β-galactosidase as described [48].

The *gftA* gene lies within the region of sequence identity between the Gifsy-1 and Gifsy-2 prophages of strain LT2 (see above) and is 100% identical to the corresponding gene in the Gifsy-2 genome of strain ATCC14028. Small ORFs initiating with UGG codons are found at the corresponding locations in ATCC14028's Gifsy-1 and Gifsy-3 prophages. These ORFs are 98% identical to each other but more distantly related to *gftA* (Figure 4A). The Gifsy-1 sequence was moved into the *ara* operon as done for *gftA* (see above). The resulting strain lysed and released high titers of phage when exposed to arabinose, consistent with the identification of this locus as an antirepressor gene. Significantly, removal of either Gifsy-1 or Gifsy-3 did not relieve the arabinose-induced lethality. Only the concomitant elimination of both prophages relieved the lethality, suggesting that the Gifsy-1 antirepressor can inactivate the Gifsy-3 repressor, GfhR, as well as GfoR. A plaque assay confirmed the presence of both phages in lysates from arabinose-treated cells (data not shown). The antirepressor genes were named GfoA (Gifsy-1) and GfhA (Gifsy-3).

### A functional *gftA* homologue in the Fels-1 genome

Derepression of Gifsy lytic transcription can also be monitored using *lacZ* fusions to a *cII* gene ortholog in the putative early right operon [10]. These fusions can be constructed by concomitantly deleting material in the right portion of the prophage (including the antirepressor gene), making it possible to test for susceptibility to antirepressors encoded by unlinked prophages (Figure S2). Thus, a *cII-lacZ* fusion that removes Gifsy-2's *gftA* gene is still MitC-inducible in an LT2 background (strain MA8363; Table 1) but not in a strain derived from ATCC14028 (MA8361). This difference might be ascribed to the presence of a duplicate copy of the *gftA* gene in the Gifsy-1 genome of LT2 and its absence in ATCC14028 (see above). Surprisingly, however, the Gifsy-2-borne *cII-lacZ* fusion remained MitC-inducible in strain LT2 after removing Gifsy-1, suggesting that yet another prophage could complement the *gftA* defect. Strain LT2 carries two other prophages, Fels-1 and Fels-2. Fels-2 seemed the most likely candidate to encode such a function in light of its strong analogies with *E. coli* phage 186, also regulated by an antirepressor mechanism [22], [24]. Unexpectedly, however, removal of Fels-1, and not Fels-2, abolished Gifsy-2 induction. To confirm the presence of a *gftA* homologue in the Fels-1 genome, an ATCC14028 strain carrying the *cII*-*lacZ* Δ*gftA* Gifsy-2 construct was lysogenized with Fels-1 phage from strain LT2. The resulting strain proved positive for *lacZ* expression when challenged with MitC. Deletion analysis localized the locus responsible for *lacZ* activation in the interval between loci STM0896 and STM0897 in Fels-1's left operon (Figure 4B). When the presumptive antirepressor gene (named *fsoA*) was placed under P^BAD^ promoter control, *lacZ* fusions to *recE* or to the *cII* ortholog in Gifsy-2 became derepressed in the presence of arabinose (Figure 4C and data not shown). The FsoA protein shares a number of amino acid identities or similarities with both GftA and GfoA (Figure 4A). Significantly, most of the GftA null mutations (see above) affect residues that are conserved in all three proteins. It seems conceivable that the most highly conserved residues (red boxes) fulfill general structural requirements for antirepressor function while identities restricted to GftA and FsoA (green boxes) might define residues contributing to the specificity of repressor recognition (Figure 4A). From the slope of the induction curves in Figure 4C, it is apparent that FsoA is less effective than GftA in relieving GftR-mediated repression. Addition of the 3xFLAG epitope sequence to the C-termini of the two antirepressor proteins does not appear to impair their activities to any significant extent (Figure 4C).

**Table 1:** Strains used in this work.

*Strain* a	*Genotype* b	*Source or reference* c
Strain LT2 derivatives		
MA6280	wild-type	[49]
TT17217	*leuD21 din-243*::MudJ	[22]
TT23381	*recN557*::MudJ *lexA33*(*lexA3* Ind^−^)::*cat*	[22]
MA7430	Gifsy-2[Δ(*int-xis*)*60*::*cat*] Gifsy-1[−]	
MA7457	Gifsy-2[Δ(*recE-recT*)*59*::*lacZ aph*] Gifsy-1[−]	[10]
MA7489	Gifsy-1[Δ(*recE-int*)*97*::*lacZ aph*] Gifsy-2[−]	[10]
MA7794	*zac-114*::*aph* (*aph* insertion on the 3′ side of *araC*)	
MA8325	Gifsy-2[Δ(*cII-sseI*)*89*::pCE36 (*lac aph*)] Gifsy-1[−]	
MA8333	Gifsy-2[Δ*124*::(*aph araC* P^BAD^)] (P^BAD^ fused to *dinI* homologue)	
MA8357	Gifsy-2[Δ*124*::(*scar* ^pSEB3^ *araC* P^BAD^) Δ(*recE-recT*)*59*::*lacZ aph* Δ(*int-xis*)*60*::*scar* ^pKD3^] Gifsy-1[−]	
MA8363	Gifsy-2[Δ(*cII-sseI*)*89*::pCE36 (*lac aph*)]	
MA8398	Fels-1[Δ(*int*-attR)*104*::*cat*]	
MA8410	Fels-1[*din-1001*::MudJ] Gifsy-1[−] Gifsy-2[−]	
MA8424	Gifsy-1[Δ(*recE-int*)*97*::*lacZ aph* Δ(*irsA-stf*)*106*::*aadA*] Fels-1[Δ(*int*-attR)*104*::*cat*] Gifsy-2[−]	
MA8425	Gifsy-1[Δ(*recE-int*)*97*::*lacZ aph* Δ(*gftA-stf*)*107*::*aadA*) Fels-1[Δ(*int*-attR)*104*::*cat*] Gifsy-2[−]	
MA8430	Gifsy-1[Δ(*recE-int*)*97*::*lacZ aph*] Fels-1[Δ(*int*-attR)*104*::*cat*] Δ(*araBAD*)*105*::*gftA-aadA* Gifsy-2[−]	
MA8456	Fels-1[*fsoR*::3xFLAG Δ(STM0897-*int*)*113*::*aph*] Δ(*araBAD*)*105*::*gftA-aadA* Gifsy-1[−] Gifsy-2[−]	
MA8508	Gifsy-1[−] Gifsy-2[−] Fels-2[−] Fels-1[Δ(*int*-attR)*104*::*cat*]	
MA8567	Δ(*araBAD*)*120*::*gftA*-3xFLAG *aph* Gifsy-1[−] Gifsy-2[−]	
MA8572	Fels-1[Δ(*int*-STM0896)*127*::*cat*]	
MA8573	Fels-1[*din-1001*::MudJ] *lexA33*(*lexA3* Ind^−^)::*cat*] Gifsy-1[−] Gifsy-2[−]	
MA8595	*zfh*-*8179*::MudJ Δ(*araBAD*)*128*::*fsoA cat*	
MA8605	Fels-1[*fsoA*::3xFLAG Δ(STM0896-*int*)*116*::*aph*] Gifsy-1[−]	
MA8728	Gifsy-2[*gftA*::3xFLAG Δ(*irsA-stf*)*114*::*scar* ^pSUB11^ *gtgR*-3xFLAG Δ(STM1011-*int*)*110*::*aph*] Fels-1[Δ(*int*-attR)*104*::*scar* ^pKD3^] Gifsy-1[−]	
MA8756	Gifsy-2[Δ(*recE-recT*)*59*::*lacZ aph* Δ(*int-xis*)*60*::*scar* ^pKD3^] Fels-1[Δ(*int*-attR)*104*::*scar*] Gifsy-1[−]	
MA8757	Gifsy-2[Δ(*recE-recT*)*59*::*lacZ aph* Δ(*int-xis*)*60*::*scar* ^pKD3^] Fels-1[Δ(*int*-attR)*104*::*scar* ^pKD3^] Gifsy-1[−] *lexA33*(*lexA3* Ind^−^)::*cat*	
MA10792	Gifsy-1[Δ(*recE-int*)*97*::*lacZ scar* ^pNFB19^] Gifsy-2[−]	
MA10796	Gifsy-1[Δ(*recE-int*)*97*::*lacZ scar* ^pNFB19^] Gifsy-2[−] Δ(*araBAD*)*105*::*gftA-aadA*	
MA10797	Gifsy-1[Δ(*recE-int*)*97*::*lacZ scar* ^pNFB19^] Gifsy-2[−] Δ(*araBAD*)*120*::*gftA*-3xFLAG *aph*	
MA10798	Gifsy-1[Δ(*recE-int*)*97*::*lacZ scar* ^pNFB19^] Gifsy-2[−] Δ(*araBAD*)*128*::*fsoA*-*cat*	
MA10799	Gifsy-1[Δ(*recE-int*)*97*::*lacZ scar* ^pNFB19^] Gifsy-2[−] Δ(*araBAD*)*121*::*fsoA*-3xFLAG *aph*	
Strain ATCC14028 derivatives		
MA5958	wild-type	[50]
MA7990	Gifsy-1[Δ*irsA108*::*aph*] Gifsy-2[−]	
MA8157	Gifsy-2[*gftR*::3xFLAG Δ(STM14_1147 - *int*)*110*::*aph*]	
MA8259	Gifsy-2[*gftR*::3xFLAG Δ(STM14_1147 -*int*)*110*::scar^SUB11^ *dinI*::3xFLAG Δ*gftA125*::*aph*] Gifsy-1[−] Gifsy-3[−] *ilvI3305*::Tn*10*dTac-*cat*-3xFLAG *aph*	
MA8327	Gifsy-2[Δ(*cII-sseI*)*89*::pCE36 (*lac aph*)] Gifsy-1[−] Gifsy-3[−] Fels-1[+]	
MA8343	Δ(*araBAD*)*99*::*gtgR*-3xFLAG *aph*	
MA8361	Gifsy-2[Δ(*cII-sseI*)*89*::pCE36 (*lac aph*)]	
MA8407	Gifsy-1[*gfoR*::3xFLAG Δ(*parA-int*)*111*::*aph*]	
MA8408	Gifsy-3[*gfhR*::3xFLAG Δ(*parA-int*)*112*::*aph*]	
MA8426	Δ(*araBAD*)*118*::*gfoR*-3xFLAG *aph*	
MA8427	Δ(*araBAD*)*119*::*gfhR*-3xFLAG *aph*	
MA8428	Δ(*araBAD*)*129*::gfhR*-3xFLAG *aph*	
MA8440	Gifsy-1[*Δ126*::(*aph araC* P^BAD^)] (P^BAD^ fused to *dinI* homologue) Gifsy-2[−] Gifsy-3[−]	
MA8468	Gifsy-2[Δ(*recE-recT*)*59*::*lacZ aph* Δ(*int-xis*)*60*::*cat*] Δ(*araBAD*)*105*::*gftA-aadA*	
MA8534	Δ(*araBAD*)*109*::*gfoA-aph* Gifsy-2[−] Gifsy-3[−]	
MA8535	Δ(*araBAD*)*109*::*gfoA-aph* Gifsy-1[−]	
MA8536	Δ(*araBAD*)*109*::*gfoA-aph* Gifsy-1[−] Gifsy-3[−]	
MA8540	Gifsy-2[Δ(*recE-recT*)*59*::*lacZ aph* Δ(*int-xis*)*60*::*scar* ^pKD3^] Δ(*araBAD*)*109*::*gfoA-scar* ^pKD13^ Gifsy-1[−] Gifsy-3[−]	
MA8541	Δ(*araBAD*)*109*::*gfoA-scar* ^pKD13^ *din-243*::MudJ Gifsy-1[−] Gifsy-3[−]	
MA8715	Gifsy-2[*gftA*::3xFLAG Δ(*irsA-stf*)*114*::*aph*]	
MA8716	Gifsy-1[*gfoA*::3xFLAG Δ(*irsA-stf*)*115*::*aph*]	
MA8725	Δ(*araBAD*)*121*::*fsoA*-3xFLAG *aph*	
MA8726	Δ(*araBAD*)*122*::*gfoA*-3xFLAG *aph*	
MA8729	Gifsy-1[*gfoA*::3xFLAG Δ(*irsA-stf*)*115*::*scar* ^pSUB11^ *gfoR*::3xFLAG Δ(*parA-int*)*111*::*aph*] Gifsy-2[−] Gifsy-3[−]	
MA8731	Δ(*araBAD*)*122*::*gfoA*-3xFLAG *aph* Gifsy-1[−] Gifsy-2[−] Gifsy-3[−]	

a All strains were derived from *Salmonella enterica* serovar Typhimurium strains LT2 [49] or ATCC14028s [50]. Most mutant alleles were constructed by the λ Red method [41]–[43]. The complete list of the oligonucleotides used as PCR primers is in Table S1.

b Square brackets following a prophage name define the genotype of that prophage. The term “scar” denotes the DNA sequence left following excision of the antibiotic-resistance cassette. Superscript indicates the plasmid used as DNA template in amplifying the cassette. For further details on phage gene nomenclature, see legend to Figure 1A. The *aph* and *aadA* genes confer resistance to kanamycin and spectinomycin, respectively. The Δ(*araBAD*)::*xxx* constructs place the gene of interest under the control of the chromosomal P^BAD^ promoter. *din-243*::MudJ and *din-1001*::MudJ denote *lacZ* transcriptional fusions (generated by transposition) to the tail operon of the Gifsy-2 prophage [22] and to the Q gene of the Fels-1 prophage (N. Figueroa-Bossi, unpublished data), respectively.

c Where not specified, the source of the strain is this work. Strains TT17217 and TT23381 were a gift of John Roth.

### Phage antirepressors accumulate in response to DNA damage

**Figure 5:**
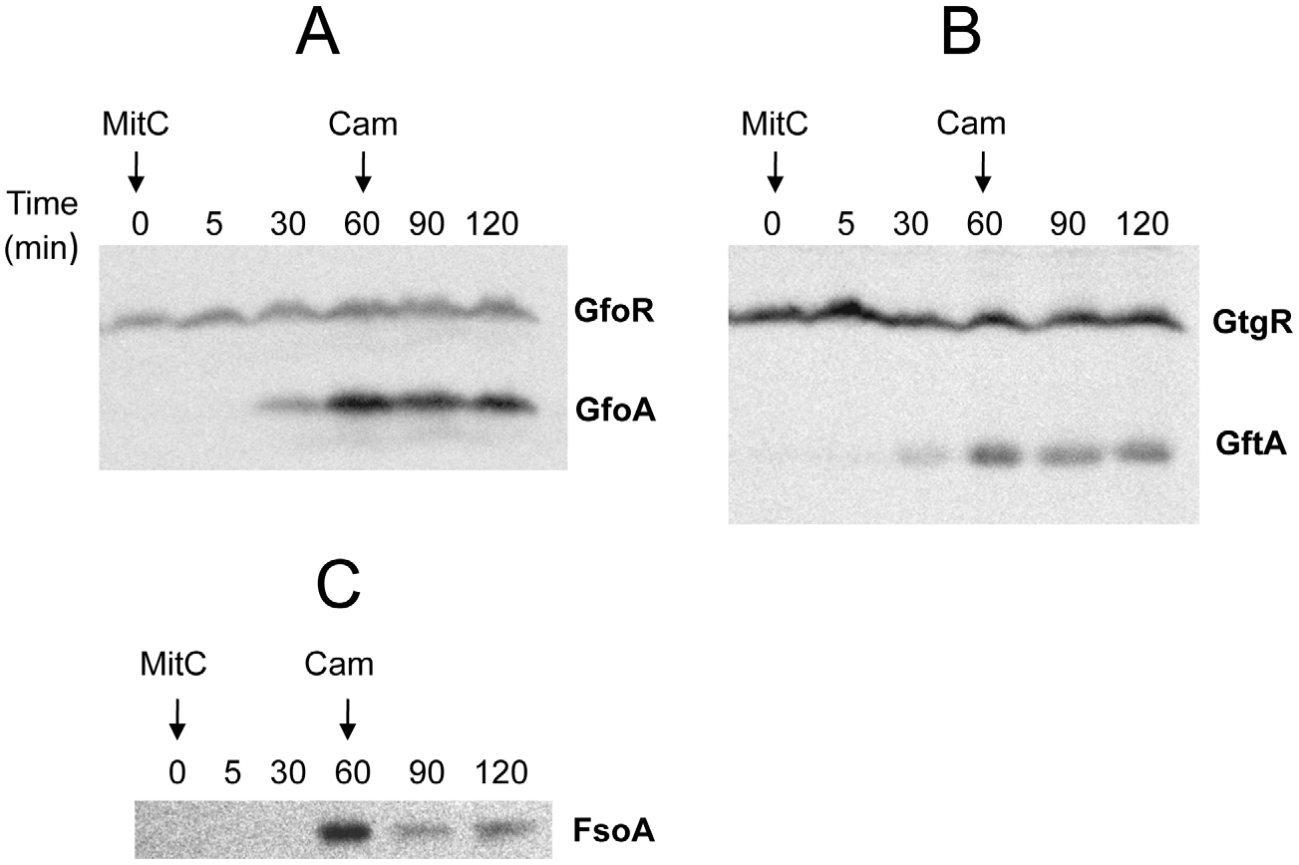
Monitoring Gifsy phage repressors and antirepressors under inducing conditions. Strains harboring 3xFLAG-tagged versions of both repressor and antirepressor genes in prophage Gifsy-1 (A; strain MA8729), Gifsy-2 (B; strain MA8728) or of the antirepressor gene in Fels-1 (C; MA8605) were exposed to Mitomycin C (1 µg mL^−1^) for 30 or 60 min. Chloramphenicol (10 µg mL^−1^) was added to samples subjected to the 60 min treatment and incubation continued for additional 30 min or 60 min. Bacteria were harvested and processed for immunodetection of epitope-tagged proteins as described [47].

Construction of epitope-tagged variants of the antirepressors allowed monitoring the regulation of these proteins by Western analysis. Figure 5 shows the results of such an experiment with cells exposed to MitC. GfoA and GftA, undetectable at the beginning of the treatment, accumulate in the presence of the drug. In contrast, as already shown in Figure 2, the levels of 3xFLAG-tagged GfoR and GftR do not change significantly throughout the treatment. Furthermore, neither the repressors nor the antirepressors were significantly affected during a one-hour chase with chloramphenicol, indicating that none of these proteins is particularly susceptible to proteolytic turnover (Figure 5). Overall, these results strongly suggest that GfoA and GftA elicit prophage induction by affecting the activity, not the concentration, of the phage repressors. Finally, the data in Figure 5 confirm that Fels-1's FsoA protein is also induced in response to DNA damage.

### Antirepressor proteins form highly stable complexes with cognate repressors

**Figure 6:**
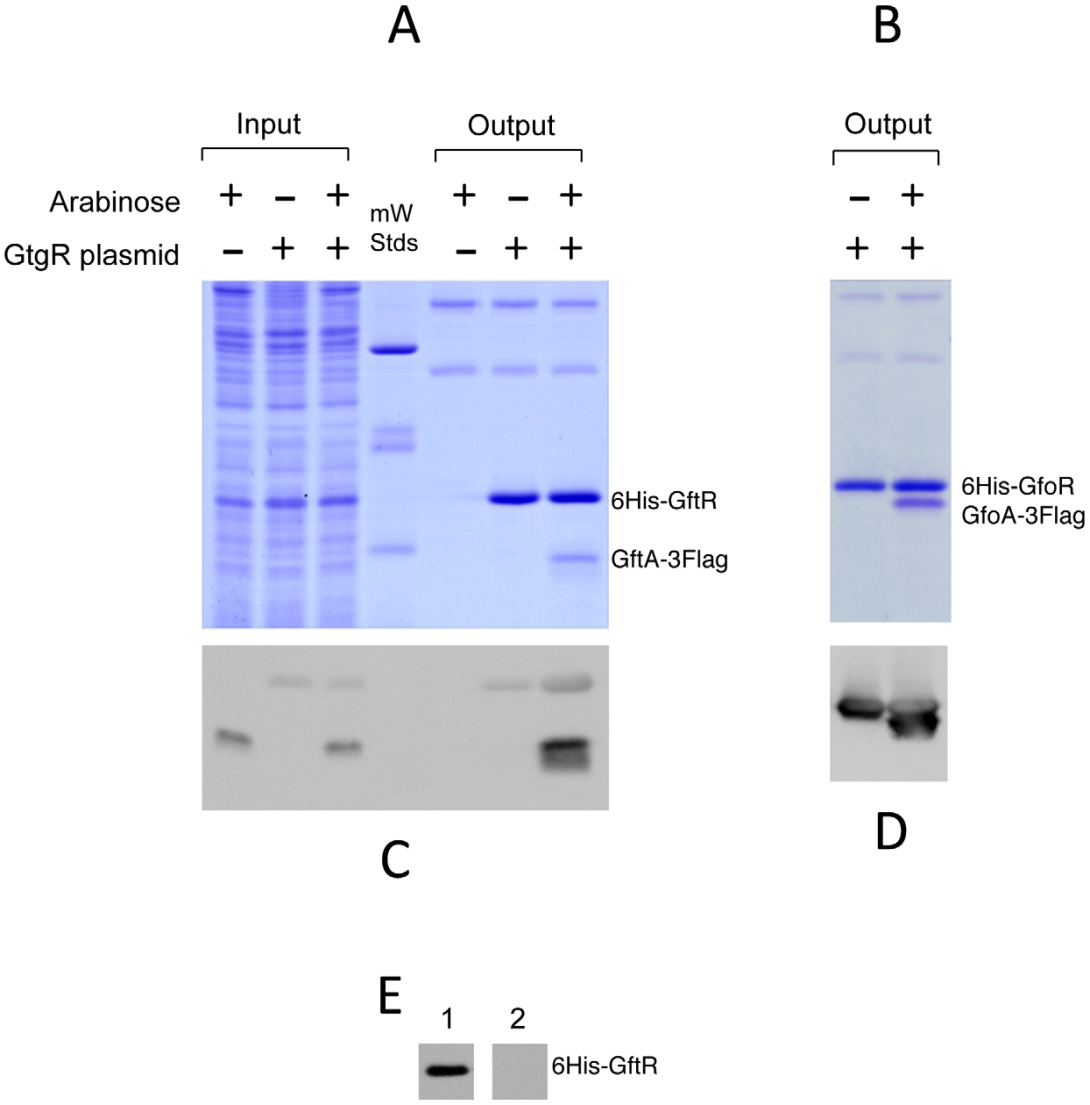
Repressor-antirepressor pulldown assays. The strains used harbor 3xFLAG-tagged versions of antirepressor genes under the control of the chromosomal P^BAD^ promoter and carry or lack plasmids expressing 6His-tagged versions of cognate repressors. Crude extracts from cells grown in the presence or absence of arabinose were incubated with nickel beads, and rinsed in low-concentration imidazole buffer (10 mM). The bound proteins were eluted from the column using high imidazole concentrations (250 mM). Eluates were applied in duplicates to 15% polyacrylamide gels and one was stained with Coomassie brilliant-blue (A and B) while the other was processed for immunodetection using anti-FLAG monoclonal antibodies (C and D). A,C. GtfA pulldown by GtgR (strain MA8567 plus or minus plasmid pSEB10; left three lanes contain samples prior to the nickel binding step); B,D. GfoA pulldown by GfoR (strain MA8731 plus or minus plasmid pSEB11). E. Far western detection of GtgR∶GftA interaction. A crude extract of strain MA8567 carrying *gtgR* plasmid pSEB10 was separated on a 15% gel and the gel blotted onto a PVDF membrane. The membrane was split into two halves; one was incubated with the extract of a strain expressing GftA-3xFLAG (1) while the other was left untreated (2). The two strips were processed for hybridization with anti-FLAG antibodies. For more details, see Materials and Methods.

The ability of Gifsy antirepressors to interact with their corresponding repressors was assessed by a surrogate pulldown assay. Strains harboring chromosomal 3xFLAG tagged antirepressor genes fused to the P^BAD^ promoter and carrying or lacking 6xHis-tagged cognate repressor genes on a plasmid, were grown in the presence or absence of arabinose. Cell-free extracts were incubated with nickel nitrilotriacetic acid agarose beads. Retained material was eluted and subjected to gel electrophoresis for direct visualization of proteins and Western blot analysis. As shown in Figure 6, in extracts from cells expressing the antirepressor genes, proteins with the molecular weight predicted for the 3xFLAG-tagged derivatives of GftA (panel A) or GfoA (panel B), were specifically retained along with the cognate repressors and revealed by the anti 3xFLAG monoclonal antibodies (panels C and D, respectively). Curiously, the anti 3xFLAG antibodies appear to react with the His-tagged repressors as well (Figure 6C). We considered that this reactivity might be due to the release of some antirepressor molecules from the membrane during the blotting procedure and their interaction with membrane-bound cognate repressors. To test this hypothesis, we asked whether antirepressor-repressor interactions could be detected by the “far Western” protocol [25]. Total proteins from a strain expressing GtgR were fractionated on an SDS gel, blotted on a Polyvinylidene fluoride (PVDF) membrane. The blot was split into two halves, one of which was incubated with a crude extract from a strain expressing 3xFLAG tagged GftA protein, prior to anti 3xFLAG antibody probing. The results in Figure 6E show that the GftR protein is only revealed in the membrane treated with the extract. This confirms that GftA and GtgR interact strongly with each other. Since the above analysis was carried out under denaturing conditions, the interaction must not require the proteins to be in their native conformation.

### Gifsy repressors and antirepressors most likely interact as dimers

**Figure 7:**
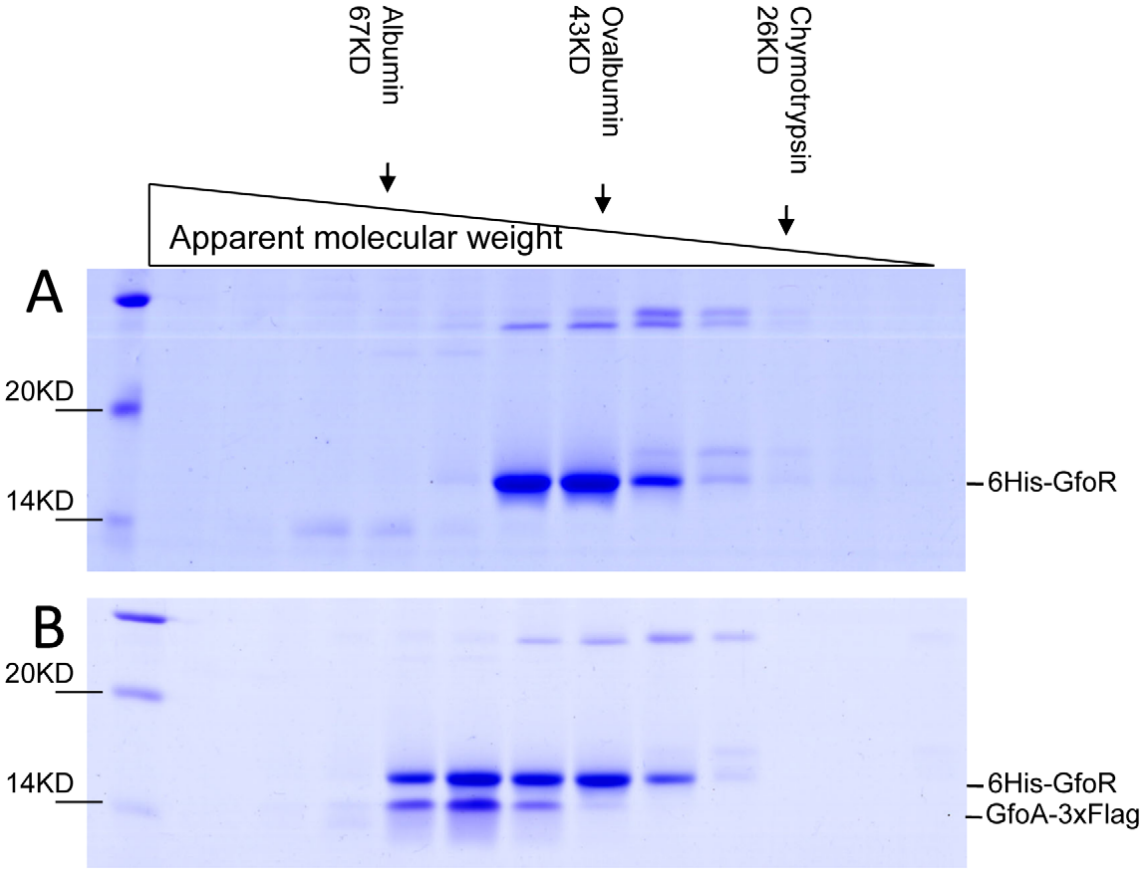
Gel exclusion chromatography of GfoR and GfoR∶GfoA complexes. Proteins were purified from strain MA8731 containing the GfoR plasmid pSEB11 as described in Materials and Methods. The strain was grown without (A) or with arabinose (B). About 10 µg of each protein preparation were applied to an Amersham Sephadex G75 column. 0.5 mL fractions were collected after the passage of the void volume, dried, resuspended in protein loading buffer and separated on a 12% SDS-polyacrylamide gel. Band identification was confirmed by anti-FLAG Western blotting (data not shown). The column was size-calibrated using α-chymotrypsin, ovalbumin and bovine serum albumin from Sigma-Aldrich.

We devoted considerable effort to determining the subunit structures of Gifsy repressors, antirepressors and their complexes by gel exclusion chromatography. This work was made difficult by a marked tendency of both proteins to form non-specific aggregates and to stick to various surfaces, particularly following buffer changes. Such problems could not be solved for the antirepressors. In contrast, using N-terminally tagged versions of the repressors, satisfactory elution profiles were eventually obtained with the repressors and their complexes. Results from a representative experiment are shown in Figure 7. GfoR and the GfoR-GfoA complex elute from a G75 Sephadex column with an apparent molecular weight of about 40 kD and 60 kD, respectively. These sizes are consistent with the GfoR being a dimer and the GfoR-GfoA complex a heterotetramer (A_2_B_2_). Similar results were obtained for the GftR/GftA complex (data not shown).

### Antirepressor binding caused cognate repressor to dissociate from the DNA

**Figure 8:**
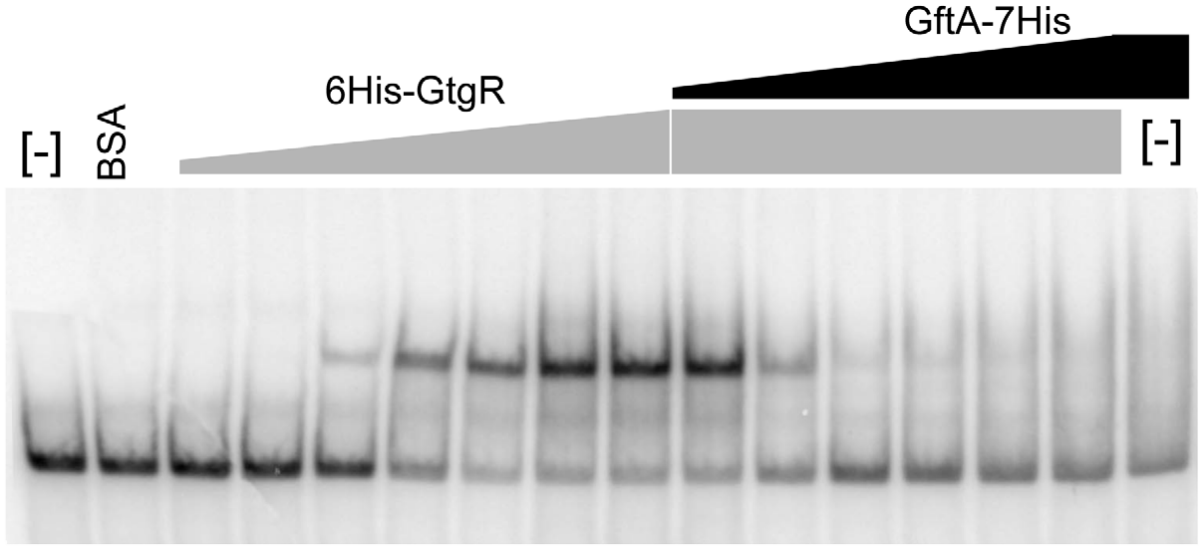
Gel-shift assay with purified GftR and GftA proteins. A radioactively labeled 267 bp DNA fragment (approximately 5 ng) was mixed with increasing amounts of purified GftR protein (∼0.25, 0.5, 1, 2, 4 or 8 pmol) in BB buffer (Tris-HCl pH 7.5 20 mM, NaCl 50 mM, EDTA 0.2 mM, MgCl2 1 mM, glycerol 5%, PMSF 10 mM, sonicated salmon sperm DNA 50 µg mL^−1^). After 15 min at room temperature, aliquots from the sample with the highest protein∶DNA ratio were mixed with increasing amounts of purified GftA protein (∼0.01, 0.05, 0.1, 0.5, 2 or 10 pmol) and incubation continued for all samples, for additional 30 min. Samples were loaded on a non-denaturing 5% polyacrylamide gel. The gel was fixed in an acid-ethanol bath, dried and radioactivity was detected and quantified by phosphorimaging.

Binding of repressors to the corresponding operator sites can be monitored by mobility shift assays in native gels. One such mobility shift is observed when a DNA fragment spanning the Gifsy-2 right operator is incubated with purified GtgR protein (Figure 8). Addition of increasing amounts of purified GftA protein to the preformed GtgR-DNA complex causes the operator fragment to be progressively released (Figure 8). Thus, these results suggest that GftA binding to the GtgR repressor causes the latter to lose affinity for DNA. No binding of GftA to DNA can be inferred from the data in Figure 8 or from a number of independent tests (data not shown). This leads us to conclude that the antirepressor most likely exerts its action by inducing a conformational change in cognate repressor, as opposed to competing for DNA binding.

## Discussion

In the present study, we have characterized the induction mechanism of the Gifsy prophages of *Salmonella*. This mechanism differs from that used by model phages λ and P22. In these phages, all information needed to elicit induction is contained within the repressor sequence. Binding of the repressors to RecA-DNA filaments formed during DNA damage, stimulates the self-catalytic proteolysis of the repressor and its inactivation. Cleavage occurs within a linker region, the “connector” that separates the N-terminal DNA-binding domain from the C-terminal dimerization domain of the protein [15]. In contrast, the regulation of Gifsy prophage induction involves two spatially separated modules: one containing the repressor gene and its sites of action (the immunity region), the other carrying a transcription unit that encodes, among others, an antirepressor protein. During normal growth, this unit is repressed by LexA, the general repressor of the SOS regulon. LexA also undergoes RecA-stimulated cleavage in the presence of damaged DNA. Antirepressor is then synthesized, it binds to and inactivates the lysogenic repressor, thereby causing the induction of the lytic program. Gifsy repressor proteins GfoR and GtgR show sequence identity with the N-terminal domain of phage λ's CI repressor for nearly their entire lengths. This suggests that these proteins lack the bipartite structure of the CI repressor and are not susceptible to self-proteolysis. A survey of the bacterial genome sequence databases reveals the existence of *gfoR*/*gtgR* homologues in prophage-like elements from a large variety of bacterial species (Figure S3). Because of their relative small size (in the 150 aa range) these proteins are unlikely to undergo self-cleavage and are thus candidates for being regulated by an antirepressor. Besides being present in the Gifsy-like prophages of many *Salmonella* enterica serovars, *gfoR*/*gtgR* homologues are found in most *Escherichia coli* strains in the database (60 genes in a total of 42 strains) as well as in *Citrobacter*, *Klebsiella*, *Yersinia* and *Enterobacter* strains (Figure S3). Two relevant members of the group are the DicA and RacR repressors of the Qin and Rac prophages respectively [26].

Previous examples of LexA-controlled antirepressors include the Tum protein of phage 186 [18] and more recently the AntC protein in phage N15 [17]. Like the GfoA and GftA proteins studied here, Tum binds to its cognate repressor and prevents its binding to the operator site [18]. Tum is nearly twice the size of the GfoA and GftA proteins (146 aa) and shows significant identity to the DinI protein in the second half of its sequence. This suggests that the antirepressor and DinI sequences are fused into a single polypeptide. Consistent with this idea, the two phage 186 relatives, *Salmonella* Fels-2 and coliphage PSP3 have the *tum* coding region split into two halves by a stop codon [22]. In phage PSP3 the upstream gene encodes the antirepressor activity and the downstream gene encodes the DinI homologue (G.E. Christie, personal communication cited in [22]). This order is reversed in the Gifsy prophages where the *dinI* homologue is the first gene of the LexA-regulated operon, followed by the antirepressor gene and by a homologue of the *irsA* gene. The DinI protein is thought to modulate the SOS response through its binding to RecA-DNA filaments; however, its exact role remains elusive [27]–[29]. This is also the case for *irsA*, a locus originally identified as the site of Tn*10* insertions impairing *Salmonella* growth in host cells (Chai and Heffron, unpublished). In the course of this study, non-polar deletions in the *dinI* or *irsA* homologues of Gifsy-1 and Gifsy-3 had no significant effects on the levels or on the rates of induction of *recE*-*lacZ* or *cII*-*lacZ* fusions (data not shown). Still, the conservation of the *dinI-irsA* region in putative prophages from *Salmonella*, *Escherichia*, *Citrobacter*, *Klebsiella* and *Enterobacter* species in genome sequence databases, suggests that these genes play some role in regulation. Overall, these findings strongly suggest that lysogenic regulation by repressor/antirepressor pairs is far more common than previously recognized. Consistent with this idea, several homologues of GfoA and GftA can be found in protein databases (Figure S4).

In the λ pathway of induction, proteolytic inactivation of the repressor makes the process irreversible. In contrast, the LexA/antirepressor-mediated mechanism can in principle be reversed if DNA damage is repaired. As LexA levels are replenished, the reduction in antirepressor synthesis will favor dissociation of the repressor-antirepressor complexes allowing the repressor to resume its function. This would probably limit viral replication and might promote reestablishment of lysogeny. One could envision the existence of a latency period during which the phage DNA undergoes limited replication before committing to the lytic pathway. The presence of chromosomal partitioning *parA* gene homologues in the left operons of the Gifsy-1 and Gifsy-3 prophages supports this idea. These properties inspire analogies with the induction pathway of *Vibrio cholerae* filamentous CTXϕ phage. In this system, LexA directly modulates the levels of the phage repressor, RstR, by activating *rstR* transcription when bound to a site overlapping with the *rstR* promoter [30], [31]. Reconstitution of the LexA pool during recovery from DNA damage was proposed to favor the reestablishment of lysogeny [31], [32]. Interestingly, RstR is the target of an antirepressor made from CTXϕ's satellite phage RS1. This protein, RstC, is not required for prophage induction; however, it is made under inducing conditions and, by inactivating RstR, is thought to prolong the RS1 and CTXϕ production period [33], [34].

The latter findings highlight an interesting property of the antirepressor function, namely its potential to serve as basis for a molecular crosstalk between phages. This feature is clearly illustrated in the present study. We found that some antirepressors can inactivate repressors made by heteroimmune prophages and trigger induction of the latter. Particularly intriguing was the discovery that the FsoA protein of the Fels-1 prophage can act on the Gifsy-2 repressor GtgR. The *fsoA* gene lies in Fels-1 left operon and is derepressed under inducing conditions following auto-proteolysis of the FsoR repressor. By targeting GtgR, FsoA effectively uncouples Gifsy-2 induction from the SOS response and puts the Gifsy-2 regulatory circuitry under FsoR control. This regulatory hijacking is difficult to rationalize since the Fels-1 prophage is induced normally in a Gifsy-2-cured strain or when the *fsoA* gene is inactivated (data not shown). However, subtle differences in induction rates or thresholds might have been missed in these experiments, and the possibility that one or more function(s) expressed from the Gifsy-2 genome positively affect(s) Fels-1 development cannot be completely ruled out. Similar effects might account for the reciprocal transactivation of Gifsy-1 and Gifsy-3 gene expression demonstrated in this study. It is also worth considering that synchronization of prophage induction in polylysogenic strains might be vital to prophages with delayed induction responses (see above). “Slow-inducing” prophages are in danger of sharing the fate of host DNA and of being destroyed when present in a strain carrying prophages that are induced more rapidly. In this scenario, paradoxically, Gifsy-2 would be the one that hijacks Fels-1 functions through FsoA.

The wide specificity of antirepressor action was first recognized in *Salmonella* phage P22. The Ant protein of P22, besides inactivating the phage's own repressor C2, can act on the repressor of Salmonella phage L and coliphages λ and 21 [35]. The role of Ant in the P22 life cycle is not completely understood. The protein is not required for induction of the P22 prophage or for any steps of the lytic or lysogenic pathways [36]. To our knowledge, the only reported activity of Ant is its ability to transactivate early gene expression in P22 lysogens when expressed constitutively from a superinfecting P22 phage [37]. It is tempting to speculate that an important role of the Ant protein is to couple induction of P22 prophage to that of other prophages in polylysogenic strains.

## Materials and Methods

### Bacterial strains and culture conditions

All strains used in this study are derivatives of *S. enterica* serovar Typhimurium. Their genotypes are listed in Table 1. The bacteria were cultured in LB broth [38] solidified by the addition of 1.5% Difco agar when needed. When appropriate, the LB medium was supplemented with 0.2% arabinose. Antibiotics (Sigma-Aldrich) were included at the following final concentrations: chloramphenicol, 10 µg mL^−1^; kanamycin monosulfate, 50 µg mL^−1^; sodium ampicillin, 75 µg mL^−1^; spectinomycin dihydrochloride, 80 µg mL^−1^; and tetracycline hydrochloride, 25 µg mL^−1^. LB plates containing 40 µg mL^−1^ 5-bromo-4-chloro-3-indolyl-β-D-galactopyranoside (X-Gal) (Sigma-Aldrich) were used to monitor *lacZ* expression in bacterial colonies. Prophages were induced using Mitomycin C (Sigma-Aldrich) at a final concentration of 1 µg mL^−1^ in liquid medium or by applying 5 µL from a 2 mg mL^−1^ stock solution on 5 mm diameter filter paper discs for plate tests. Liquid cultures were grown in New Brunswick gyratory shakers, and growth was monitored by measuring the optical density at 600 nm with a Milton-Roy Spectronic 301 spectrophotometer.

### Genetic techniques

Generalized transduction was carried out using the high frequency transducing mutant of phage P22, HT 105/1 *int-201* [39]. Typically, P22 lysates were used at a 1∶50 dilution, mixed with aliquots from overnight cultures of recipient bacteria in a 1∶2 ratio, and incubated for 30 min at 37°C prior to being plated on selective media. Transductant colonies were purified by two sequential passages on selective plates and verified to be free of phage by streaking on Evans Blue Uranine plates [40]. Chromosomal engineering was carried out by the λ Red recombination method [41]–[43] as previously described [20]. Donor DNA fragments were generated by PCR using plasmid or chromosomal DNA templates. A complete list of the oligonucleotides used as primers in these experiments is in Table S1. Amplified fragments were electroporated into appropriate strains harboring the conditionally replicating plasmid pKD46, which carries a λ *red* operon under the control of the P^BAD^ promoter [41]. Bacteria carrying pKD46 were grown at 30°C in the presence of ampicillin and exposed to arabinose (10 mM) for 3 hours prior to preparation of electrocompetent cells. Electroporation was carried out using a Bio-Rad MicroPulser under the conditions specified by the manufacturer. Recombinant colonies were selected on LB plates containing the appropriate antibiotic. Constructs were verifed by PCR and/or DNA sequencing. When needed, the antibiotic resistance cassette was excised by transforming strains with plasmid pCP20, which encodes the Flp recombinase [44].

### Plasmids

Plasmids used as PCR templates for λ Red-mediated gene disruptions included pKD3, pKD4 and pKD13 [41]. Plasmid pSUB11 was the template in the construction of 3x-FLAG epitope fusions [20]. Additional plasmid templates constructed in the present work were pSEB1, which carries the spectinomycin-resistance *aadA* cassette, and pSEB3, carrying an *aph*-*araC*-P^BAD^ module. For the construction of pSEB1, the *aadA* gene of plasmid pBT22 [45] was amplified by PCR with primers pp411 and pp412 (Table 2); the resulting fragment was digested with EcoRI and ligated into EcoRI-cleaved plasmid pSUB2. The latter is a derivative of pGP704 [46] lacking the BamHI segment spanning the RP4 *tra* operon. Plasmid pSEB3 was derived from a chromosomal construct carrying the kanamycin-resistance *aph* gene immediately downstream from *araC* gene (strain MA7794; Table 1 and Table S1). The *aph*-*araC*-P^BAD^ segment was amplified from MA7794 chromosomal DNA with primers le41 and le42 (Table 2), cleaved with EcoRI, and cloned into pSUB2.

**Table 2:** DNA oligonucleotides used as PCR primers for plasmid construction and cloning.

Primer^a^	Sequence (5′– 3′)
pp411	TTTCCTCTTCCTTCTCTCGAATTC ***ACCTTGCCGTAGAAGAACA***
pp412	TTTCCTCTTCCTTCTCTCGAATTC ***TTTGGCTGTGAGCAATTATG***
le41	CTTCTCTCCCTCCTCCTCCGAATTC ***ACCCCGTCCCCCTTCGTC***
le42	CCTCCTCCTCCCTCTCTTCTGAATTC ***CATCGTCTTACTCCATCCAG***
le144	AATTAGTCGACAGGAGGAGGACGTTCATGCACCACCATCATCACCAT***AAAGAAAAAACTCATCAGATTAAT***
le145	TATTAATATTATATATTACGGCCGT***TACTCCGAGCTTTTATCTTAA***
le146	AATTAGTCGACAGGAGGAGGACGTTCATGCACCACCATCATCACCAT***AACAAAAATCTTCATCCCAT***
le147	TATTAATAATATATATACGGCCG ***TTACTATTTTTTGAGGTCGTTAATT***
pp849	CTAGAAGGAGATATACCATGGGCCACCACCACCACCACCACCACTCTCGAGCTCCACCACCACCACCACCACCACTAGTAAG
pp850	TCGACTTACTAGTGGTGGTGGTGGTGGTGGTGGAGCTCGAGAGTGGTGGTGGTGGTGGTGGTGGCCCATGGTATATCTCCTT
pp864	ATATAATAATTAAATATAAACTCGAGCCA***TGG*** ***CAGAGGGAGTCCTATCA***
pp865	ATATTTAATTATCAAACTAGTGGAGCTCA***TTATTAGAGCCCATCTCTCTGAC***
pp866	ATATAATAATTAAATATAAACTCGAGTCA***TGA*** ***GTAATTCAGCTTTGCAA***
pp867	ATATTTAATTATCAAACTAGTGGAGCTC ***TATATCAGAAGGTGGTGTTACC***

a Relevant restriction enzyme cleavage sites are underlined. The sequences annealing to template DNA are shown in bold italics.

A second set of recombinant plasmids was constructed to overproduce and purify phage repressor and antirepressor proteins. Repressor genes *gftR* and *gfoR* were amplified from wild-type ATCC14028 chromosomal DNA with primer pairs le146/le147 and le144/le145, respectively (Table 2). In both cases, the forward primer contained a 5′ extension designed to produce an N-terminal 6xHis tag fusion. The amplification products were doubly digested with SalI and EagI restriction endonucleases and ligated to SalI×EagI-cleaved pKTQ12 DNA [45] yielding plasmids pSEB10 (*gftR*) and pSEB11 (*gfoR*). A different vector, pNFB28, was used for cloning antirepressor genes. Plasmid pNFB28 is a derivative of Novagen's pET-16b plasmid modified so as to allow the construction of both N-terminal and C-terminal 7xHis tag fusions. The modification involved ligating a DNA fragment produced by annealing oligonucleotides pp849 and pp850 (Table 2) to pET-16b DNA doubly digested with XbaI and XhoI endonucleases. *gftA* and *gfoA* genes were amplified from wild-type ATCC14028 chromosomal DNA with primer pairs pp864/pp865 and pp866/pp867, respectively. The amplified fragments were doubly digested with NcoI and SacI (*gftA*), and BspHI and SacI (*gfoA*), and ligated to pNFB28 DNA digested with NcoI and SstI. In the resulting plasmids, pSEB12 and pSEB13, the *gftA* and *gfoA* genes carry 7xHis-encoding sequences at their 3′ ends and are under the control of the T7 promoter.

### Protein purification

For the purification of repressors and repressor/antirepressor complexes, strains MA8567 (P^BAD^-*gftA*-3xFLAG) and MA8731 (P^BAD^-*gfoA*-3xFLAG), carrying or lacking plasmids pSEB10 and pSEB11, respectively, were grown to an OD_600_≈0.15 at 37°C and exposed to 0.1% L-arabinose (Sigma-Aldrich) or left untreated. Bacteria were cultivated at 37°C for additional 6 hours, Cells were harvested by centrifugation at 10000G and rinsed once in PBS (137 mM NaCl, 2.7 mM KCl, 100 mM Na_2_HPO_4_ and 2 mM KH_2_PO_4_). Pellets were then submitted to several freeze-thaw cycles in a dry-ice/ethanol bath before being resuspended in IP buffer (Tris-HCl 20 mM pH 8, NaCl 500 mM, Igepal 0.1% (Sigma-Aldrich), imidazole 20 mM) and sonicated on ice until complete but gentle lysis. Cell debris was spun down and the supernatant applied to Nickel nitrilotriacetic acid (Ni-NTA) resin (Qiagen). Incubation was continued for 2 hrs at 4°C. Liquid was removed and the resin was rinsed twice with 10 times the extract volume of buffer IP. Proteins still specifically bound to the resin were eluted in buffer IPE identical to buffer IP except for imidazole concentration (250 mM). Fractions were then adjusted to 15% glycerol and frozen at −80°C for storage. The purity as assessed from the repressor content was greater than 80% and concentration was in the range of 0.1–1 µg mL^−1^.

C-terminally 7xHis tagged GftA protein was purified from *E. coli* strain BL21 carrying plasmid pSEB12. Cells were grown essentially as described above but induction was carried out with IPTG (0.1 mM final concentration) for 3 hrs. Bacterial pellets were processed as described above, except that Igepal was omitted from IP and IPE buffers.

### Size-exclusion chromatography

A Sephadex G75 column (Amersham) previously calibrated with α-chymotrypsin, ovalbumin and bovine serum albumin purchased from Sigma-Aldrich was connected to an Äkta P-9000 HPLC apparatus and a Frac-950 fraction collector. To eliminate any aggregate that might have formed during storage, protein samples were systematically centrifuged at maximum speed in a micro centrifuge for 15 min prior to loading. About 10 µg of proteins were loaded onto a column pre-equilibrated with about 2 volumes of IPE buffer. After the passage of the void volume, 0.5 mL fractions were collected and vacuum dried. Samples were resuspended in loading buffer, boiled and separated on a 12% SDS-polyacrylamide gel.

### Western blot analysis

Western blotting was conducted essentially as previously described [47]. Briefly, bacteria from 2 ml overnight cultures were harvested by centrifugation and resuspended in 50–80 µL of Laemmli buffer. Cells were lysed by boiling 10 min and lysates loaded onto 15% SDS-polyacrylamide gels. Biorad's Precision Plus Kaleidoscope standards were included as migration markers. After the gel run, proteins were electro-transferred to a PVDF membrane, which was blocked with PBS containing 3% skimmed milk and 0.05% Tween 20. The blocking buffer was then replaced with a similar buffer containing the primary anti-FLAG antibody (anti-FLAG M2 from Sigma-Aldrich) for 30 min. The membrane was rinsed thoroughly in PBS 0.05% Tween 20 before the secondary antibody (anti-mouse peroxidase-labeled secondary antibodies from Sigma-Aldrich) was applied. Finally, results were revealed with the ECL kit from Amersham and imaged on a Fuji LAS3000 apparatus.

### Gel-shift assays

A DNA fragment spanning the binding site of the GftR was amplified by PCR from strain LT2 chromosomal DNA with primers le127 (5′-GTTCGCCGATGCTCATTT-3′) and le128 (5′-CCGTGAGAGGTCAGCCATA-3′). The PCR product was then radioactively labeled with T4 polynucleotide kinase (NEB) and γ-^32^P-ATP as recommended by the manufacturer. Labeled DNA (approximately 5 ng) was mixed with 5 µL of 5× buffer BB (Tris-HCl pH 7.5 100 mM, NaCl 250 mM, EDTA 1 mM, MgCl_2_ 5 mM, glycerol 25%, PMSF 250 mM sonicated salmon sperm DNA 250 µg mL^−1^) and varying amounts of protein. The reaction volume was adjusted with water to a final reaction volume of 20 µL. When appropriate, GftA antirepressor was added to GftR∶DNA complexes formed during an initial 15 min incubation at room temperature. Incubation was continued at the same temperature for additional 30 min (for samples with or without added GftA). Samples were loaded onto a 5% non-denaturing polyacrylamide gel. After electrophoretic separation, gels were fixed in 20% ethanol 10% acetic acid, dried, and imaged with a Storm 820 apparatus from Molecular Dynamics.

## Supporting Information

Figure S1 Analysis of *gfhR* expression patterns. The *gfhR* gene contains two functional in-frame AUG initiation codons. DNA segments corresponding to the long (gfhR*) and the to short (*gfhR*) open reading frame (including a C-terminal 3xFLAG tag) were fused to the chromosomal P^BAD^ promoter by recombineering techniques. The resulting strains, MA8427 (A) and MA8428 (B) were grown in the absence or in the presence of arabinose, lysed, and processed for Western blot analysis. Detection of high levels of GfhR protein in construct A in the absence of arabinose suggests that the interval between the two initiating AUGs contains a promoter element.
(TIF)

Figure S2 Relevant constructs used in this study. A. Structure of Gifsy-2 prophage in strains MA8756, MA8757, MA8468 and MA8540. B. Structure of Gifsy-2 prophage in strains MA8325, MA8327, MA8361 and MA8363. C. Structure of Gifsy-1 prophage in strains MA8424 (Δ*106*) and MA8425 (Δ*107*). D. Structure of Gifsy-1 prophage in strain MA7990. Genes marked by an asterisk are named on the basis of their sequence similarity to known genes of other phages or bacteria.
(TIF)

Figure S3 Sequence alignement of proteins with homology to the GfoR repressor (A) and to the GftR repressor (B). Protein database were searched using the BlastP program with a cut-off -value of <10^−3^. Hits originating from *Salmonella* sequences were omitted. Conservation is expressed as shades of red with a darker color corresponding to better conservation. No highlight indicate <50% conservation.
(TIF)

Figure S4 Sequence alignement of proteins with homology to the GfoA antirepressor (A) and to the FsoA antirepressor (B). Data were obtained and processed as described in the legend to Figure S3. Hits originating from *Salmonella* sequences were omitted.
(TIF)

Table S1 DNA oligonucleotides used as PCR primers in λ Red-mediated constructions.
(DOC)
