# Supplementary figures and images for: Bacteriophage Crosstalk: Coordination of Prophage Induction by Trans-Acting Antirepressors
Source: PLoS Genet. 2011 Jun 23;7(6):e1002149. doi: 10.1371/journal.pgen.1002149 (PMC3121763; doi:10.1371/journal.pgen.1002149)

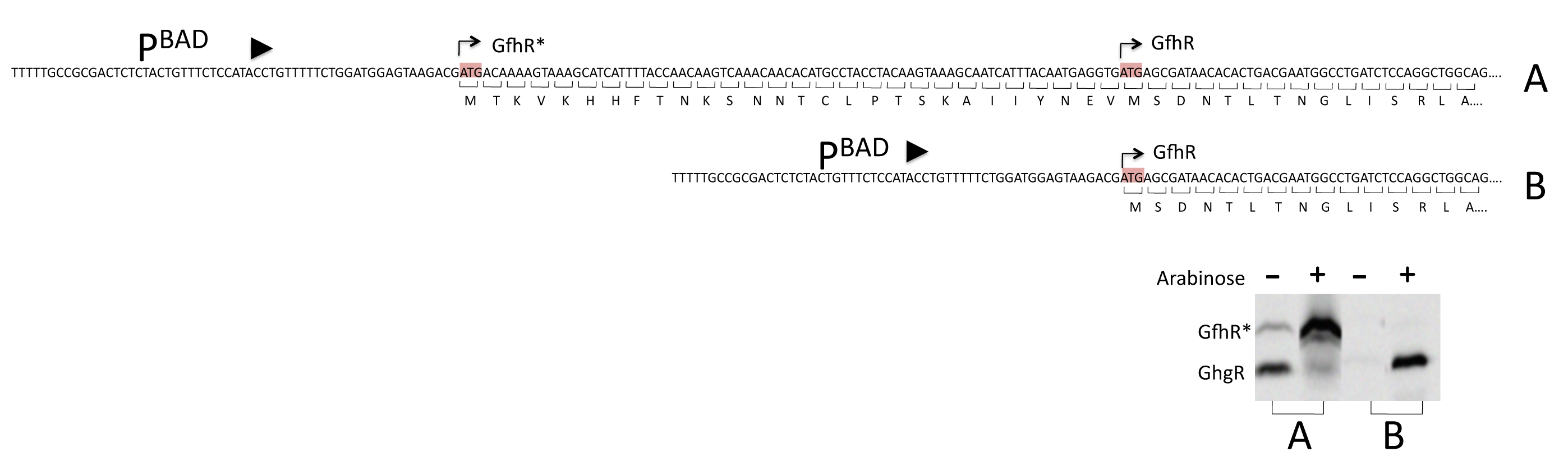

Supplement: Figure S1 — Analysis of gfhR expression patterns. The gfhR gene contains two functional in-frame AUG initiation codons. DNA segments corresponding to the long (gfhR*) and the to short (gfhR) open reading frame (including a C-terminal 3xFLAG tag) were fused to the chromosomal PBAD promoter by recombineering techniques. The resulting strains, MA8427 (A) and MA8428 (B) were grown in the absence or in the presence of arabinose, lysed, and processed for Western blot analysis. Detection of high levels of GfhR protein in construct A in the absence of arabinose suggests that the interval between the two initiating AUGs contains a promoter element. (TIF) [file pgen.1002149.s001.tif]

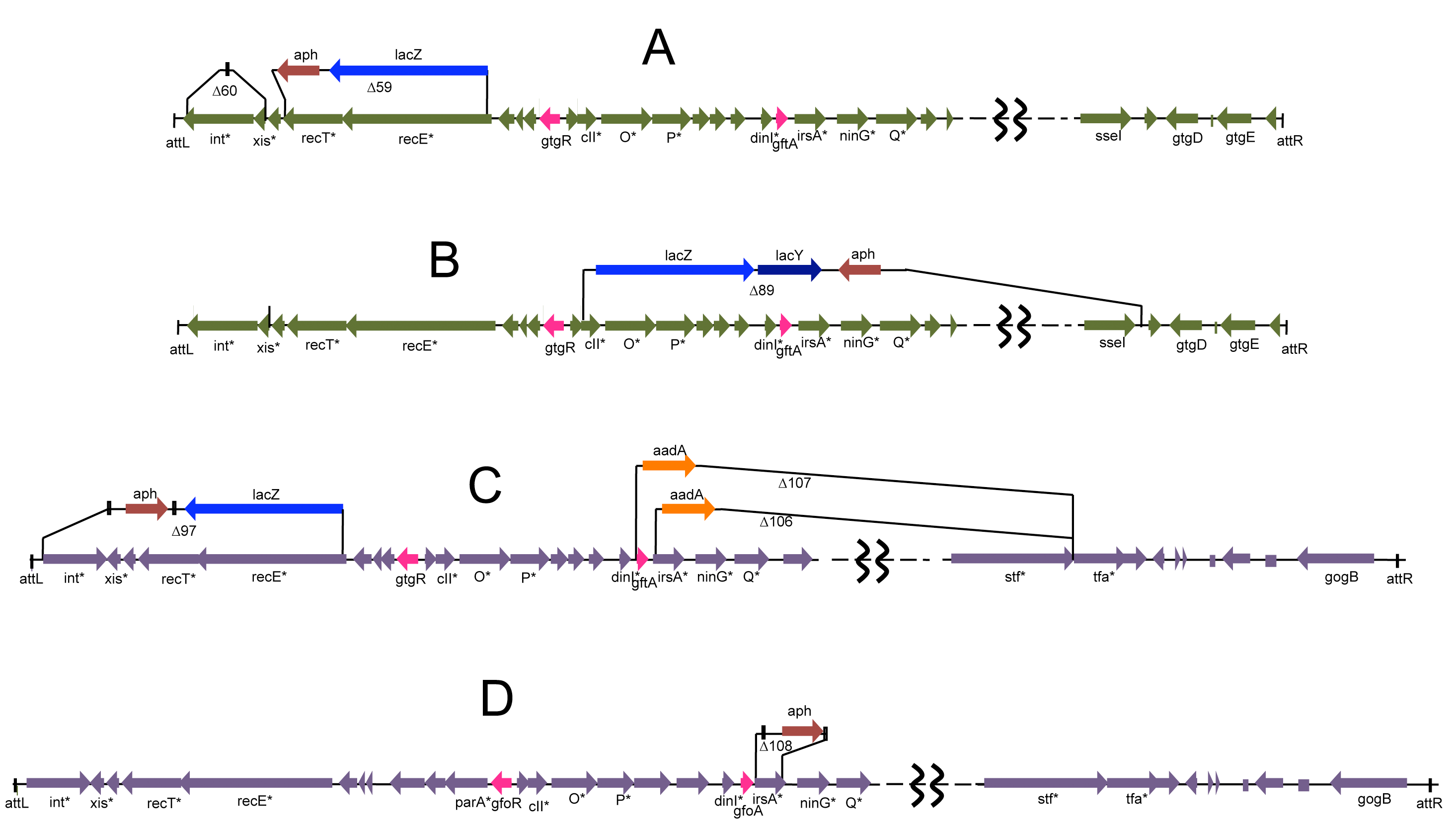

Supplement: Figure S2 — Relevant constructs used in this study. A. Structure of Gifsy-2 prophage in strains MA8756, MA8757, MA8468 and MA8540. B. Structure of Gifsy-2 prophage in strains MA8325, MA8327, MA8361 and MA8363. C. Structure of Gifsy-1 prophage in strains MA8424 (Δ106) and MA8425 (Δ107). D. Structure of Gifsy-1 prophage in strain MA7990. Genes marked by an asterisk are named on the basis of their sequence similarity to known genes of other phages or bacteria. (TIF) [file pgen.1002149.s002.tif]

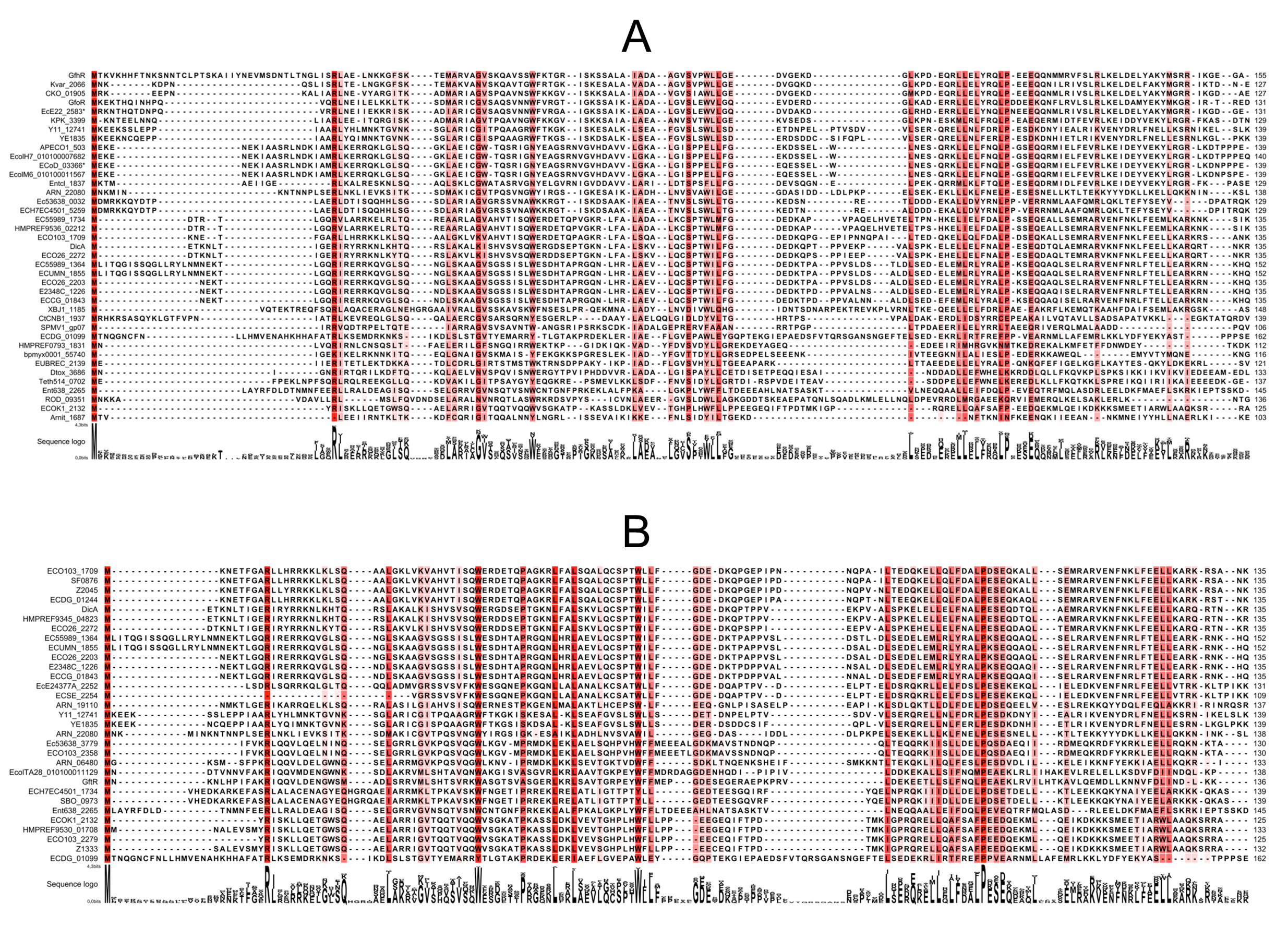

Supplement: Figure S3 — Sequence alignement of proteins with homology to the GfoR repressor (A) and to the GftR repressor (B). Protein database were searched using the BlastP program with a cut-off -value of <10−3. Hits originating from Salmonella sequences were omitted. Conservation is expressed as shades of red with a darker color corresponding to better conservation. No highlight indicate <50% conservation. (TIF) [file pgen.1002149.s003.tif]

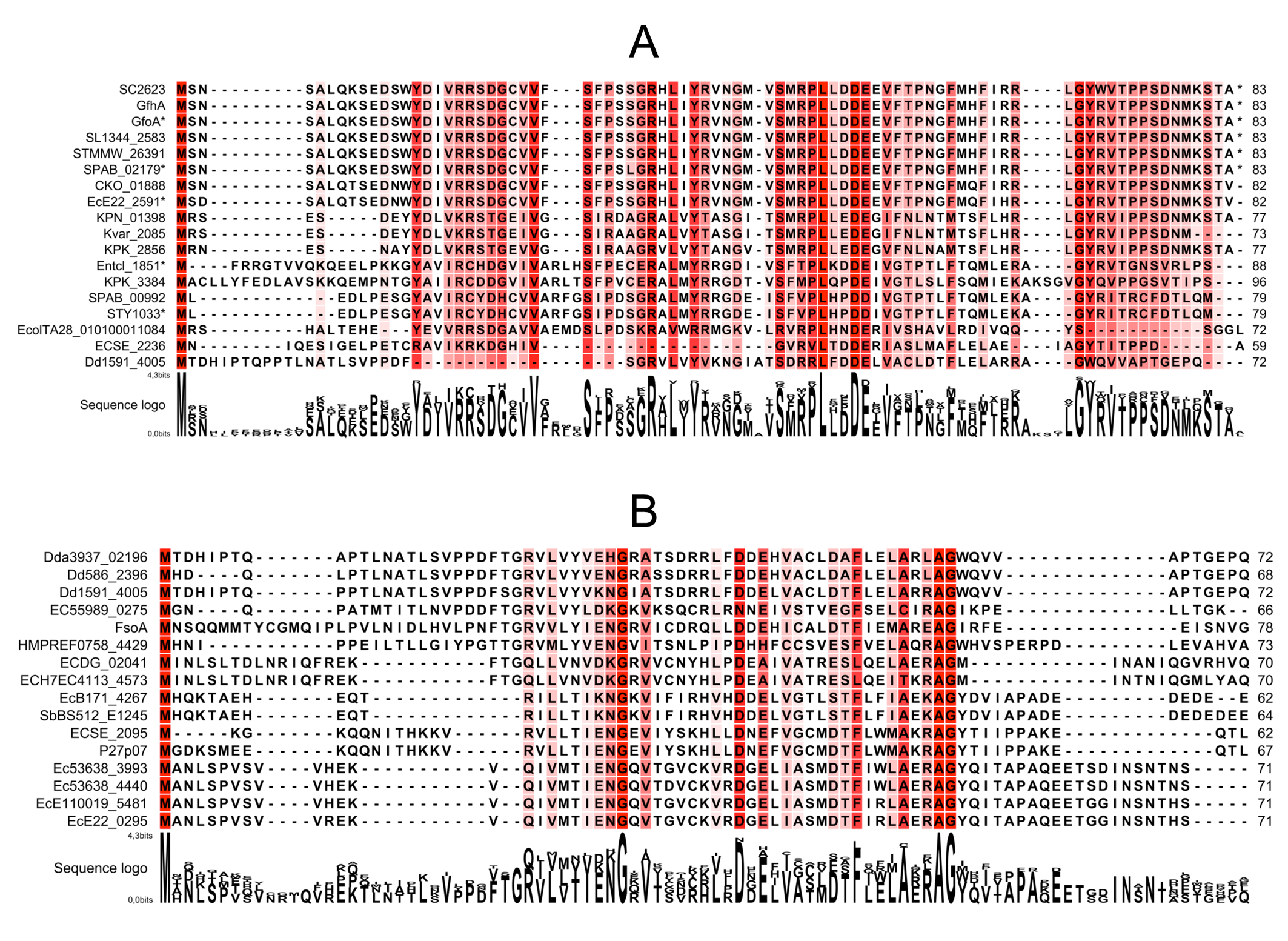

Supplement: Figure S4 — Sequence alignement of proteins with homology to the GfoA antirepressor (A) and to the FsoA antirepressor (B). Data were obtained and processed as described in the legend to Figure S3. Hits originating from Salmonella sequences were omitted. (TIF) [file pgen.1002149.s004.tif]
