# Supplementary material for: Bacteriophage Crosstalk: Coordination of Prophage Induction by Trans-Acting Antirepressors
Source: PLoS Genet. 2011 Jun 23;7(6):e1002149. doi: 10.1371/journal.pgen.1002149 (PMC3121763; doi:10.1371/journal.pgen.1002149)
Supplement: Table S1 — DNA oligonucleotides used as PCR primers in λ Red-mediated constructions. (DOC) [file pgen.1002149.s005.doc]

**Table S1.** **DNA oligonucleotides used as PCR primers in λ Red-mediated constructions**

| Primer^a^ | Sequence (5’– 3’) | Template | Allele |
| --- | --- | --- | --- |
| le39(l)  le40 (r) | AAAGTGAATGATGTGTCCACAAAGATGTCATAAGCCGTAA***ATTCCGGGGATCCGTCGACC*** ACCCCGTCCCCCTTCGTCAAGCCGTCAATTATCCGAATAG***TGTAGGCTGGAGCTGCTTCG*** | pKD13 | *zac*-*144*::*aph* |
| pp199 (l)  pp198 (r) | TATCGTCCAGGGTGACGCGGTACTTATCGTGCAGTTCGTC***GCTCTGCCAGTGTTACAACC***  CATCAGGCCAGAAAGACGCTGTACTCTGGTGCAGTGATG***ccgtcgttttacaacgtcgtg*** | pUT lacZ | Gifsy-2 (LT2) ∆[*recE-recT*]*59*::*lacZ* *aph* |
| pp321 (l)  pp198 (r) | CATCAGGCCAGAAAfGACGCTGTACTCTGGTGCAGTGATG***ccgtcgttttacaacgtcgtg*** TTTGTGTCCACCAGTGCGTCCATATCCTATGATGGACACA***ATGTAGGCTGGAGCTGCTTC*** | pNFB19 | Gifsy-1 (LT2) ∆[*recE-int*]*97*::*lacZ* *aph* |
| pp286 (l)  pp285 (r) | CCTGTTCTGCGCTCTTCTCTGACATCCACTTTCCATACAC***CATATGAATATCCTCCTTAG***  CGATGAGCAACATTATCCAACTGACGCCAAACAAGTGGGT***TGTAGGCTGGAGCTGCTTCG*** | pKD3 | Gifsy-2 (LT2) ∆[*int-xis*]*60*::*cat* |
| le91 (l) le92 (r) | ATAACCACTTAACATCTTGTTTTATCTAAATAAAATTAAG***CATATGAATATCCTCCTTAG***  AATCATACGAAAATCGTCCAAGAGCATATGATTTTCAGTA***TGTAGGCTGGAGCTGCTTCG*** | pKD3 | Fels-1 ∆[*int*-attR]*104*::*cat* |
| le13 (l) pp140 (r) | CCTCACGAGATTGACCCAGCAGCATACCCAAACCCAACCG***TGTGTAGGCTGGAGCTGCTTC***  AAGAAAACCACCTCACCCTCATAACTCAGTAAGCGTCCCG***CATATGAATATCCTCCTTAG*** | pKD3 | Gifsy-2 (LT2) ∆[*cII-sseI*]*89*::*cat* |
| le136 (l)  le139 (l) | ATAACCACTTAACATCTTGTTTTATCTAAATAAAATTAAG***TGTAGGCTGGAGCTGCTTCG***  GTAAACGTGAAATTCAAAAATGGAATGAGAAATACGATGC***CATATGAATATCCTCCTTAG*** | pKD3 | Fels-1∆[*int*-STM0896)*127*::*cat* |
| le106 (l)  pp641 (r) | ACAACCAGTAACCTGACAATTATGCGCCACGGAGAATACC***GATCATCGAGCTCTCCCGG*** TTAACCGTGGTTTCCGCATTACCGTCTGCGTCCACAATAA***CGGCTTGAACGAATTGTTAG*** | pSEB1 | Gifsy-1 (LT2) ∆[*irsA-stf*]*106*::*aadA* |
| le107 (l) pp641 (r) | GCAGGAAACGTGGGAAAGCGCTGACGACTGGTTTTATTAA***GATCATCGAGCTCTCCCGG*** TTAACCGTGGTTTCCGCATTACCGTCTGCGTCCACAATAA***CGGCTTGAACGAATTGTTAG*** | pSEB1 | Gifsy-1 (LT2) ∆[*gftA-stf*]*107*::*aadA* |
| pp644 (l)  pp654 (r) | GAATACCATGGCGCACGAATTACAACTCATCAAGCAGTCA***GATCCGTCGACCTGCAGTTC***  ATTTATACAACTGCTGAAATTCGACCTCATCCATGCTGGA***TGTGTAGGCTGGAGCTGCTT*** | pKD13 | Gifsy-1 (14028) ∆*irsA108*::*aph* |

**Table S1**, continued

| Primer | Sequence (5’– 3’) | Template | Allele |
| --- | --- | --- | --- |
| le60 (l) le61 (r) | GAAGAACAACGTTTTTGATCTAATTAACGACCTCAAAAAA***GACTACAAAGACCATGACGG***  TCTGCGCTCTTCTCTGACATCCACTTTCCATACACCTTGTT***CATATGAATATCCTCCTTAG*** | pSUB11 | Gifsy-2 (14028) *gftR*::3xFLAG  ∆[*parA*-*int*]*110*::*aph* |
| pp341 (l) pp342 (r) | TCTGCAGGAAACGTGGGAAAGCGCTGACGACTGGTTTTAT***GACTACAAAGACCATGACGG***  ATCTCTCTGACAACTTCCATAATGGTATCCTTAGACCAGT***CATATGAATATCCTCCTTAG*** | pSUB11 | Gifsy-2 (14028) *dinI*::3xFLAG ∆*gftA125*::*aph* |
| le102(l)  le101 (r) | TCTTCCGTTCTGATGGACACATGCAGGGATAAATCATGGC***CATATGAATATCCTCCTTAG***  ACGAGAAGTACATGAAGGGAAGGATTCGAACGCGCGAAGAT***GACTACAAAGACCATGACGG*** | pSUB11 | Gifsy-1 (14028) *gfoR*::3xFLAG  ∆[*parA-int*]*111*::*aph* |
| le104 (l)  le103 (r) | CCAAACTTTCCCCAAAACGTTTCCCCAAAACCCTTATGA***CATATGAATATCCTCCTTAG***  CGCGAAGTACATGAGCCGCAGGATAAAAGGTGAAGGCGCA***GACTACAAAGACCATGACGG*** | pSUB11 | Gifsy-3 (14028) *gfhR*::3xFLAG  ∆[*parA-int*]*112*::*aph* |
| le123 (l)  le122 (r) | ATAACCACTTAACATCTTGTTTTATCTAAATAAAATTAAG***CATATGAATATCCTCCTTAG***  GGTGTTACTTAGCCAATCTCAACAAATTCGACGCCACGCC***GACTACAAAGACCATGACGG*** | pSUB11 | Fels-1 *fsoR*::3xFLAG  ∆[STM0897*-int*]*113*::*aph* |
| le124 (l)  pp890 (r) | TACCATTATGGAAGTTGTCAGAGAGATGGGCTCTAATAAT***GACTACAAAGACCATGACGG***  TTAACCGTGGTTTCCGCATTACCGTCTGCGTCCACAATAA***CATATGAATATCCTCCTTAG*** | pSUB11 | Gifsy-2 (14028) *gftA*::3xFLAG  ∆[*dinI-stf*]*114*::*aph* |
| le163 (l)  pp890 (r) | TCGTCGTCTCGGCTACCGGGTAACACCACCTTCTGATATA***GACTACAAAGACCATGACGG***  TTAACCGTGGTTTCCGCATTACCGTCTGCGTCCACAATAA***CATATGAATATCCTCCTTAG*** | pSUB11 | Gifsy-1 (14028) *gfoA*::3xFLAG  ∆[*dinI-stf*]*115*::*aph* |
| le162 (l) le123 (r) | CGAAGCAGGAATCCGTTTTGAGGAGATATCAAATGTTGGA***GACTACAAAGACCATGACGG***  ATAACCACTTAACATCTTGTTTTATCTAAATAAAATTAAG***CATATGAATATCCTCCTTAG*** | pSUB11 | Fels-1 *fsoA*::3xFLAG  ∆[STM0896*-int*]*116*::*aph* |
| pp753 (l) pp751 (r) | TTTGCCTAAAATGTGATATGAAACAACACACTAGCCTTTG***ATGTAGGCTGGAGCTGCTTC***  AATTCCCTCTACATTCAACCACTGTATATAAACACAGTAT***CATCGTCTTACTCCATCCAGAA*** | pSEB3 | Gifsy-2 (LT2) ∆*124*::[*aph araC* P^BAD^]-*dinI* |
| pp754 (l) pp752 (r) | TTTCCGCCTAAAATCTGATATGAAACAACATGCTAGCTTT***ATGTAGGCTGGAGCTGCTTC*** AATTCCCTCTACATTTAACTACTGTATATAAACACAGTAT***CATCGTCTTACTCCATCCAGAA*** | pSEB3 | Gifsy-1 (14028) ∆*126*::[*aph araC* P^BAD^]-*dinI* |

**Table S1**, continued

| Primer | Sequence (5’– 3’) | Template | Allele |
| --- | --- | --- | --- |
| le37 (l) le38 (r) | ACTGTTTCTCCATACCTGTTTTTCTGGATGGAGTAAGACG***ATGAACAAAAATCTTCATCCCAT***  TGCTGCATGTCGGGCAACTGCGGCGCAAGCTGGCGGCAGA***CCACTTTCCATACACCTTGTT*** | MA8279 | ∆[*araBAD*]*99*::*gftR*-3xFLAG *aph* |
| le117 (l) le115 (r) | CTGTTTCTCCATACCTGTTTTTCTGGATGGAGTAAGACGA***TGAGTAATTCAGCTTTGCAAAA*** TTTGCGTAGATAGTGTTTATCCAGCAGGGATTGCTGCATG***TGTGTAGGCTGGAGCTGCTT*** | MA8377 | ∆[*araBAD*]*109*::*gfoA-aph* |
| le108 (l) le109 (r) | ACTGTTTCTCCATACCTGTTTTTCTGGATGGAGTAAGACG***ATGAAAGAAAAAACTCATCAG***  TGCTGCATGTCGGGCAACTGCGGCGCAAGCTGGCGGCAGA***CATATGAATATCCTCCTTAG*** | MA8407 | ∆[*araBAD*]*118*::*gfoR*-3xFLAG *aph* |
| le116 (l)  pp501 (r) | GTTTCTCCATACCTGTTTTTCTGGATGGAGTAAGACGA***TGGCAGAGGGAGTCCTATC*** TTTGCGTAGATAGTGTTTATCCAGCAGGGATTGCTGCATG***CGGCTTGAACGAATTGTTAG*** | MA8424 | ∆[*araBAD*]*105*::*gftA-aadA* |
| le110 (l) le109 (r) | ACTGTTTCTCCATACCTGTTTTTCTGGATGGAGTAAGACG***ATGACAAAAGTAAAGCATCAT***  TGCTGCATGTCGGGCAACTGCGGCGCAAGCTGGCGGCAGA***CATATGAATATCCTCCTTAG*** | MA8408 | ∆[*araBAD*]*119*::*gfhR*-3xFLAG *aph* |
| le111 (l) le109 (r) | ACTGTTTCTCCATACCTGTTTTTCTGGATGGAGTAAGACG***GTGATGAGCGATAACACACT***  TGCTGCATGTCGGGCAACTGCGGCGCAAGCTGGCGGCAGA***CATATGAATATCCTCCTTAG*** | MA8408 | ∆[*araBAD*]*129*::*gfhR**-3xFLAG *aph* |
| le154 (l) le156 (r) | GTTTCTCCATACCTGTTTTTCTGGATGGAGTAAGACG***ATGAACTCACAGCAAATGATGA***  TGCTGCATGTCGGGCAACTGCGGCGCAAGCTGGCGGCAGA***TGTAGGCTGGAGCTGCTTCG*** | MA8572 | ∆[*araBAD*]*128*::*fsoA*-*cat* |
| le116 (l)  le119 (r) | GTTTCTCCATACCTGTTTTTCTGGATGGAGTAAGACGA***TGGCAGAGGGAGTCCTATC***  TTTAGAGGCATTACTGCCCGTAATAGGCTTTTGCGCCGTG***CATATGAATATCCTCCTTAG*** | MA8715 | ∆[*araBAD*]*120*::*gftA*-3xFLAG *aph* |
| le154 (l) le119 (r) | GTTTCTCCATACCTGTTTTTCTGGATGGAGTAAGACG***ATGAACTCACAGCAAATGATGA***  TTTAGAGGCATTACTGCCCGTAATAGGCTTTTGCGCCGTG***CATATGAATATCCTCCTTAG*** | MA8605 | ∆[*araBAD*]*121*::*fsoA*-3xFLAG *aph* |
| le175 (l) le119 (r) | GTTTCTCCATACCTGTTTTTCTGGATGGAGTAAGACGA***TGAGTAATTCAGCTTTGCAAAAG***  TTTAGAGGCATTACTGCCCGTAATAGGCTTTTGCGCCGTG***CATATGAATATCCTCCTTAG*** | MA8716 | ∆[*araBAD*]*122*::*gfoA*-3xFLAG *aph* |

^a^ Primers are defined as "left" (l) or "right" (r) based on the orientation of the prophage map or of the *ara* operon structural genes. The portions of primers annealing to template DNA are shown in bold italics.
